# Transcriptomic and metabolomic shifts in rice roots in response to Cr (VI) stress

**DOI:** 10.1186/1471-2164-11-648

**Published:** 2010-11-20

**Authors:** Sonali Dubey, Prashant Misra, Sanjay Dwivedi, Sandipan Chatterjee, Sumit K Bag, Shrikant Mantri, Mehar H Asif, Arti Rai, Smita Kumar, Manju Shri, Preeti Tripathi, Rudra D Tripathi, Prabodh K Trivedi, Debasis Chakrabarty, Rakesh Tuli

**Affiliations:** 1 National Botanical Research Institute, Council of Scientific and Industrial Research, Rana Pratap Marg, Lucknow 226 001, India; 2 National Agri-Food Biotechnology Institute (Department of Biotechnology, New Delhi), Mohali, Punjab, India

## Abstract

**Background:**

Widespread use of chromium (Cr) contaminated fields due to careless and inappropriate management practices of effluent discharge, mostly from industries related to metallurgy, electroplating, production of paints and pigments, tanning, and wood preservation elevates its concentration in surface soil and eventually into rice plants and grains. In spite of many previous studies having been conducted on the effects of chromium stress, the precise molecular mechanisms related to both the effects of chromium phytotoxicity, the defense reactions of plants against chromium exposure as well as translocation and accumulation in rice remain poorly understood.

**Results:**

Detailed analysis of genome-wide transcriptome profiling in rice root is reported here, following Cr-plant interaction. Such studies are important for the identification of genes responsible for tolerance, accumulation and defense response in plants with respect to Cr stress. Rice root metabolome analysis was also carried out to relate differential transcriptome data to biological processes affected by Cr (VI) stress in rice. To check whether the Cr-specific motifs were indeed significantly over represented in the promoter regions of Cr-responsive genes, occurrence of these motifs in whole genome sequence was carried out. In the background of whole genome, the lift value for these 14 and 13 motifs was significantly high in the test dataset. Though no functional role has been assigned to any of the motifs, but all of these are present as promoter motifs in the Database of orthologus promoters.

**Conclusion:**

These findings clearly suggest that a complex network of regulatory pathways modulates Cr-response of rice. The integrated matrix of both transcriptome and metabolome data after suitable normalization and initial calculations provided us a visual picture of the correlations between components. Predominance of different motifs in the subsets of genes suggests the involvement of motif-specific transcription modulating proteins in Cr stress response of rice.

## Background

Chromium (Cr) is the seventh most abundant metal in the earth's crust and an important environmental contaminant released mainly by leather, paint and fertilizer industries [1,2]. Stable forms of Cr are the trivalent Cr (III) and the hexavalent Cr (VI) species. Being a strong oxidizer, Cr (VI) is highly toxic and more mobile in soil/water systems than Cr (III) [3]. Cr contaminates food sources and accumulates in agricultural products through water, air, and polluted soils posing a serious health risk to people worldwide [4]. Rice consumers are exposed to significant amounts of chromium and other heavy metals dissolved in field water [5]. In reduced soil environment of rice crop, the heavy metals go into soil solution, risking their uptake by the rice crop [4,6,7]. The WHO/FAO expert committee on food additives has set the provisional maximum tolerable weekly intake for Cr at 23.3 μg/Kg^-1^ of body wt week^-1^[7,8].

Several studies have been carried out on the uptake and translocation of Cr from soil by different plants [5]. Cr (VI) is actively taken up by active processes, probably mediated by carriers of essential ions such as sulfate or iron, whereas Cr (III) is probably taken up passively by cation exchange [3]. Chromium compounds are detrimental to seed germination, seedling growth, growth and development, leading to severe oxidative damage to cells [9-11]. Chromium interferes with several metabolic processes including photosynthesis, water relation and uptake of nutrients, resulting in reduced root growth and phytomass, chlorosis, stunting and finally plant death [5,9,12]. Panda [11] reported the effect of Cr exposure on roots of developing rice seedlings and concluded that the induction of oxidative stress is the main process underlying Cr toxicity in plants.

In plants, a predominant model for detoxification of heavy metals is by the formation of complexes of heavy metals with phytochelatins (PCs). As a detoxification mechanism, such complexes are compartmentalized in vacuoles. However, unlike other heavy metals such as Cu, Pb and Cd, Cr is unable to induce PCs, and thus detoxification mechanism for this metal is poorly understood [3,10]. Molecular events underlying Cr toxicity and the defense signal transduction have been only partially elucidated. A number of genes potentially involved in Cr resistance and accumulation were identified by cDNA-AFLP [13]. The study suggested four willow species (*Salix alba, Salix elegans, Salix fragilis *and *Salix matsudana*) had the existence of common mechanisms of gene regulation in response to Cr, pathogen attack and senescence-mediated programmed cell death. However, no mechanism specific to Cr was identified.

Transcriptional regulation, also known as transcriptome reprogramming, is essential for plant adaptation to biotic and abiotic stresses. Use of "omic" studies have provided more inside about the physiological and molecular effects of environmental stress which led to the identification of genes involved and their expression patterns during the course of stress perception and response [14]. Recent studies suggested that signaling pathways regulated by abscisic acid, salicylic acid, jasmonic acid and ethylene, as well as ROS signaling pathways, play key roles in the crosstalk between biotic and abiotic stress signaling. The molecular mechanisms that are involved in each stress have been revealed independently. However, understanding of convergence points between biotic and abiotic stress signaling pathways remain rudimentary [15]. Therefore, it is necessary to explore the cross talk between the abiotic-stress responses.

High-throughput genomic technologies have made it possible to analyze expression of thousands of genes at a time. We have earlier reported comparative genome-wide transcriptome analysis of rice seedlings treated with As(III) and As(V) and identified molecular processes and networks associated with response to these metals [16]. Detailed analysis of genome-wide transcriptome profiling in rice root is reported here, following Cr-plant interaction. Such studies are important for the identification of genes responsible for tolerance, accumulation and defense response in plants with respect to Cr stress. Rice root metabolome analysis was also carried out to relate differential transcriptome data to biological processes affected by Cr (VI) stress in rice. Our analyses suggest that Cr-specific motifs identified in this study are significantly over represented in the promoter regions of Cr-responsive genes.

## Results and discussion

### Physiological responses of rice seedling to chromium stress

Seedlings grown on media containing different concentrations of Cr (VI) showed retardation in the growth of the seedlings with increasing concentration of Cr (VI). The shoot and root growth in terms of root length, shoot length, root and shoot weight was significantly inhibited at 100 μm Cr (VI) with respect to control. Significant decline (40-50%) in the length and weight of roots (Table 1) was observed in seedlings exposed to 100 μM Cr. Our results are in accordance to the reported Cr toxicity on seedling growth, nutrient and water imbalance [11].

**Table 1:** Effect of Cr (VI) on root and shoot length and biomass of rice plant (IR-64)

Parameters	0 μM	25 μM	50 μM	100 μM	250 μM
Shoot Length (mm)	23.8 ± 0.273	22.8 ± 0.447	22.2 ± 0.570	17.4 ± 0.547**	17.2 ± 0.447**

Root Length (mm)	7.3 ± 0.836	6.9 ± 0.741	6.6 ± 0.547*	6.5 ± 0.655*	5.8 ± 0.447**

Shoot FW (mg)	160.95 ± 0.636	134 ± 0.282*	90.8 ± 0.282**	58.5 ± 0.282***	47.4 ± 0.565***

Shoot DW (mg)	27.45 ± 0.63	26.55 ± 0.424	24.2 ± 0.848*	15.15 ± 0.494***	14 ± 0***

Root FW (mg)	52.25 ± 0.353	48.5 ± 0.707	41.6 ± 0.565*	32 ± 0.707***	26.2 ± 0.282***

Root DW (mg)	3.91 ± 0.296	3.35 ± 0.353	2.43 ± 0.240**	2.345 ± 0.360**	1.95 ± 0.494***

Shoot (Conc. Cr accumulation mg kg^-1 ^dw)	00	46.84 ± 4.05*	211.95 ± 50.91**	679.76 ± 133.70**	1298.80 ± 146.37***

Root (Conc. Cr accumulation mg kg^-1 ^dw)	00	994.31 ± 21.99***	1963.03 ± 465.94***	2560.77 ± 150.80***	4106.58 ± 29.58***

The values are means of triplicates ± SD. *, ** and *** = significantly different at P ≤ 0.05, P ≤ 0.01 and P ≤ 0.001, respectively, according to Student's unpaired t-test.

**Figure 1:**
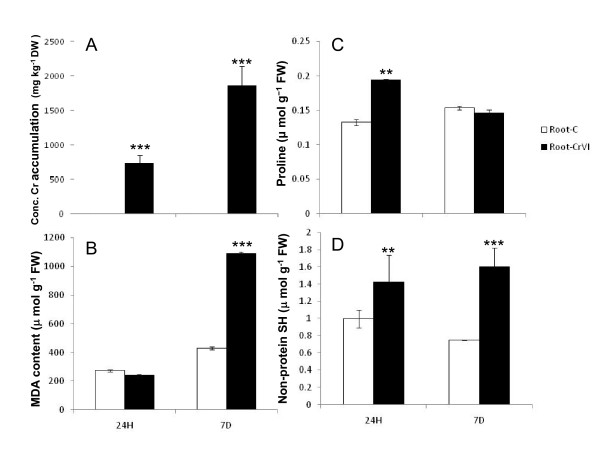
**Effect of 100 mM Cr (VI) on the level of Cr accumulation (A), MDA (B), Proline (C), Non-protein thiol (D) in rice root after 24 h and 7 days treatment**. The rice variety IR-64 was germinated and allowed to grow for 5 d at 37°C and then transferred to Hewitt solution for growth. After 10 d of growth, seedlings of uniform size and growth were treated with Cr (VI) (100 μM) under standard physiological conditions of 16 h light (115 μmol m^-2 ^s^-1^) and 8 h dark photoperiod at 25 ± 2°C. The values are means of triplicates ± SD. ** and *** = significantly different at P ≤ 0.01 and P ≤ 0.001, respectively, according to Student's unpaired t-test.

Chromium accumulation in roots and shoots increased with increasing concentration of Cr (VI). The chromium accumulation was higher in roots as compared to shoot at all the concentrations (Table 1). After 24 h the root tissue accumulated 735 mg kg^-1 ^DW Cr. At 25 μM Cr exposure, roots accumulated more that 20 fold Cr in comparison to shoots. This difference in Cr accumulation in both the tissues decreased with increased Cr exposure. At 100 μM, roots accumulated only 2 fold higher Cr in comparison to shoot. The results suggest that at higher concentration roots mobilize Cr to shoot affectively to minimize Cr toxicity in roots. Cr was not detected in any part of the control plants (Figure 1A).

Significant increase in lipid peroxidation was observed after 7 d treatment (Figure 1B). MDA, a decomposition product of polyunsaturated fatty acid hydroperoxides, has often been utilized as a biomarker for lipid peroxidation [17-19], which is an effect of oxidative damage. Loss of membrane integrity is the final and irreversible phase of cell damage and is closely linked with membrane lipid peroxidation [20]. Large changes in the physical properties of membrane lipids during heavy-metal stress appear to contribute to the loss of membrane's selective permeability.

Free proline levels in roots were higher in 24 h treatment in comparison to 7 d post treatment (Figure 1C). Rapid accumulation of free proline is a typical response to heavy metal stress [21]. When exposed to abiotic and biotic stress, many plants accumulate high amounts of proline, in some cases several times the sum of other amino acids [22]. Proline protects cell membranes of onion against salt injury [23] and is generally assumed to serve as a physiologically compatible solute that maintains a favorable osmotic potential between the cell and its surroundings. After 7 d, the oxidative injury was much higher as evidenced by higher level of free radical-induced peroxidation of lipid membranes. Proline level declined as it was unable to protect the cell from oxidative damage.

The level of non-protein thiols was statistically higher in roots after 24 h and 7 d treatment with 100 μM Cr (Figure 1D). Their function is attributed to heavy metal detoxification and the homeostasis of essential nutrients through the immobilisation of metal ions and their subsequent vacuolar sequestration, thus preventing them from interfering with cellular metabolism [17,20]. Thiol incorporation is made at the cost of sulphur-containing proteins necessary for development. This may retard overall growth of the plant as seen in our study (Table 1). The stable amount of thiols in control root results from a balance between the input and the output of non-protein thiols, through the incorporation of cystein. Exposure to Cr disturbs this balance because of the high consumption of these thiol groups.

### General features of Cr (VI)-stress expression profiles

Roots are the main organ for heavy metal penetration. We exposed roots of 7 d old seedlings for 24 h in 100 μM Cr (VI) to analyze the change in transcriptome. The higher metal exposures yielded a linear increase in root tissue accumulation, leading to higher degree of toxicity. However, our results evidenced the importance of the concentrations used in works of this nature, since in exposures to environmentally relevant concentrations, which are themselves representative of high contaminations; plants were able to cope with stress without displaying any noticeable phytotoxic effects, despite some accumulation of the metal accumulation. However, short-term exposure to higher Cr concentration indicated the vulnerability of this species to high Cr. Genome wide gene expression analysis was conducted using RNA from rice roots exposed to Cr (VI) (100 μm) for 24 h in hydroponic solution. An average of 47% probe sets hybridize to the transcripts, as analyzed by GCOS. Utilizing high-density Affymetrix gene-chip based microarray representing 51,279 genes, we observed that the expression of 1138 genes was up-regulated, and that of 1610 genes was down-regulated by Cr (VI). Functional analysis of these genes was done to relate the genome response to the shift in biological activities. The differentially expressed genes belong to various gene families related to transporters, cellular stress response, regulatory proteins, growth and development and secondary metabolism. A list of the up- and down-regulated probe sets under Cr stressed rice seedling roots with their annotations are provided in Additional files 1 and 2, Tables S1 and S2, respectively.

Oligonucleotide primers for the 22 genes related to transport, stress, and defense functions were used for validation of the microarray data through RealTime PCR (Table 2). The validation was carried out separately for root, to resolve tissue-specific differences in the expression of selected genes. The results obtained from the 22 genes tested by real time PCR agree with the trend of regulation identified by microarray analysis. The PDR-like ABC transporter gene (Os07g33780) was highly expressed to Cr in root, two multidrug resistance protein 4 (Os01g50100, Os04g13210) were expressed at a higher level in roots, five glutathione S-transferase GSTU6 (Os01g37750, Os10g38350, Os10g38495, Os10g38610, Os01g72150), two peptide transporter (Os06g03560, Os04g50940) and one sulfate transporter 3.5 (Os01g52130) were specifically up-regulated in chromium-stressed roots. This validates the microarray analysis. Three HSPs (Os03g16920, Os05g38530, and Os03g16030) and seven cytochrome P450 (Os01g41810, Os02g36030, Os01g43740, Os03g12500, Os01g41820, Os03g55240, Os06g45960) genes were up-regulated in the chromium treated root as in case of the microarray results.

**Table 2:** Comparison of microarray and RT-PCR analysis of selected genes, differentially regulated during Cr (VI) stress in rice

Locus ID	Description	Microarray (Log2)	RT-PCR (Log2)
LOC_Os07g33780	PDR-like ABC transporter, putative, expressed	14.07	33.59 ± 0.25

LOC_Os08g30770	ABC transporter, ATP-binding protein, putative, expressed	9.3	12.84 ± 3.31

LOC_Os01g50100	ABC transporter, ATP-binding protein, putative, expressed	57.54	93.05 ± 0.009

LOC_Os04g13210	Multidrug resistance-associated protein 4, putative, expressed	22.92	20.11 ± 0.06

LOC_Os06g03560	Oligopeptide transporter 9, putative, expressed	3.63	3.83 ± 0.08

LOC_Os04g50940	Peptide transporter PTR2, putative, expressed	9.39	3.83 ± 0.24

LOC_Os01g52130	Sulfate transporter 3.5, putative, expressed	13.22	16.45 ± 0.04

LOC_Os01g41810	Cytochrome P450 72A1, putative, expressed	71.1	139.10 ± 0.08

LOC_Os02g36030	Cytochrome P450 76C2, putative, expressed	41.12	448.82 ± 0.08

LOC_Os01g43740	Cytochrome P450 72A1, putative, expressed	39.85	80.45 ± 0.15

LOC_Os03g12500	Cytochrome P450 74A2, putative, expressed	30.15	120.26 ± 0.002

LOC_Os01g41820	Cytochrome P450 72A1, putative, expressed	26.32	182.28 ± 0.2

LOC_Os03g55240	Cytochrome P450 81E1, putative, expressed	23.35	87.43 ± 0.18

LOC_Os06g45960	Cytochrome P450 CYP99A1, putative, expressed	22.74	171.25 ± 0.27

LOC_Os01g37750	Glutathione S-transferase GSTU6, putative, expressed	74.43	347.29 ± 0.56

LOC_Os10g38350	Glutathione S-transferase GSTU6, putative, expressed	16.85	4.96 ± 0.03

LOC_Os10g38495	Glutathione S-transferase GSTU6, putative, expressed	15.23	74.02 ± 0.19

LOC_Os10g38610	Glutathione S-transferase GSTU6, putative, expressed	12.61	15.24 ± 0.03

LOC_Os01g72150	Glutathione S-transferase, putative, expressed	14.75	30.06 ± 0.13

LOC_Os03g16920	Heat shock cognate 70 kDa protein (DnaK family protein), putative, expressed	63.42	2683.69 ± 12.4

LOC_Os05g38530	Heat shock cognate 70 kDa protein (DnaK family protein), putative, expressed	12.71	5.43 ± 0.81

LOC_Os03g16030	17.4 kDa class I heat shock protein 3, putative, expressed	12.56	7.88 ± 0.09

### Gene annotation and detail gene response to Cr (VI)-stress

**Figure 2:**
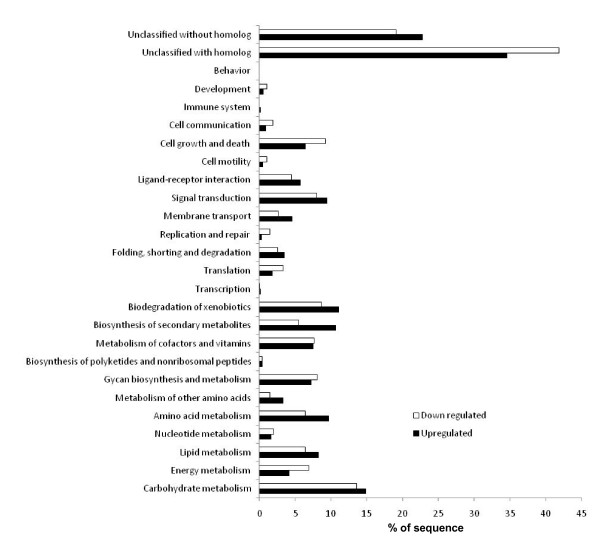
**Functional groups of differentially expressed genes following Cr (VI) stress**. The rice variety IR-64 was germinated and allowed to grow for 5 d at 37°C and then transferred to Hewitt solution for growth. After 10 d of growth, seedlings of uniform size and growth were treated with different concentrations of Cr (0 and 100 μM) under standard physiological conditions. Total RNA was extracted from the treated rice roots using and microarray was performed using one-cycle target labeling and control reagents. The hybridization data were analyzed using dCHIP software. For the genes that were differentially regulated, the gene ontology classification provided in http://bioinfoserver.rsbs.anu.edu.au/utils/GeneBins/ was used to assign genes to a hierarchical biological process using the Genebins.

Functional distribution of the submitted probe sets (Up-regulated and down-regulated) in the 2nd level of the GeneBins ontology is provided in Figure 2http://bioinfoserver.rsbs.anu.edu.au/utils/GeneBins/. The percentage represents the proportion of submitted probe sets that have been assigned in the corresponding functional category. Many genes are indicated as "unclassified" because the annotation of the rice genome does not assign any putative function and these are stated as ''hypothetical" or ''expressed". A large set of differentially expressed genes belongs to the defense and stress response in plants, in addition to being involved in general metabolism. The current analyses showed that 1138 genes were up-regulated, and 1610 genes were down-regulated by Cr (VI). Among the down-regulated genes, the most affected genes are for energy metabolism (111 genes), carbohydrate metabolism (218), stilbene, coumarine and lignin biosynthesis (60), phenylalanine metabolism (53), cell growth and death (150), photosynthesis (26), lipid metabolism (103), biodegradation of xenobiotics (140), photosystem II (15), amino acid metabolism (103), cell cycle (46) etc.

Among the up-regulated genes, the most affected genes are biosynthesis of secondary metabolites (122), specially flavonoid biosynthesis (58), lipid metabolism (95), amino acid metabolism (111), carbohydrate metabolism (170), biodegradation of xenobiotics (127), ascorbate and aldarate metabolism (42), membrane transport (53) specially ABC-2.A ABC-2 type transport system ATP-binding protein (11) and ABC transporters (17), glutathione metabolism (18), MAPK signaling pathway (75) and a large number of glutathione S-transferase (18) etc. On percentage basis, the most effected genes that are down-regulated during Cr (IV) stress are those for energy metabolism, cell growth and death, development. The most up-regulated genes are related to amino acid metabolism, biosynthesis of secondary metabolites and xenobiotics, membrane transport and signal transduction.

**Figure 3:**
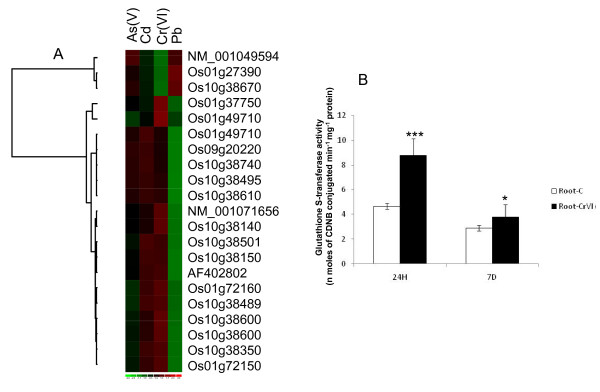
**Modulation in expression and activity of glutathione-S- transferase during Cr-stress**. Expression profiles of differentially expressed GSTs after Cr (VI), As (V), Cd and Pb stress in rice root (A). The rice variety IR-64 was germinated and allowed to grow for 5 d at 37°C and then transferred to Hewitt solution for growth. After 10 d of growth, seedlings of uniform size and growth were treated with 100 μM of Cr (VI), As (V), Cd, and Pb under standard physiological conditions. Total RNA was extracted from the treated rice roots and microarray was performed using one-cycle target labeling and control reagents. The hybridization data were analyzed using dCHIP software. The color scale (representing log signal values) is shown at the bottom. Effect of 100 μM Cr (VI) on the level of GST-activity (B) in rice root after 24 h and 7 days treatment.

The genes coding for proteins such as glutathione S- transferase (GSTs; eighteen genes; Os01g37750, Os09g20220, Os10g38350, Os10g38495, Os01g72150, Os10g38610, Os01g49710, Os01g49710, Os10g38600, Os10g38600, 001071656, Os10g38140, Os01g72160, Os10g38740, Os10g38501, Os10g38150, AF402802, Os10g38489) which are involved in xenobiotic metabolism, are found to be unregulated in Cr stress. GSTs are known to be induced by a number of intracellular and environmental factors that include heavy-metal stress [17]. Normally, GSTs catalyze the conjugation of toxic molecules with reduced glutathione (GSH) and target them for ATP-dependent transport into the vacuole. The reduced (GSH)/oxidized (GSSG) glutathione ratio increases steadily through the course of the experiment under chromium stress [24]. This indicates that GSH was rapidly oxidized to GSSG indicating a possible role of GSH as an antioxidant in chromium stress in plants. Standeven and Wetterhahn [25] reported that GSH protects against the acute nephrotoxicity of Cr(VI), although it is not clear whether GSH is directly involved in the intracellular metabolism of Cr(VI) at non-toxic doses. We analyzed expression of GSTs in various vegetative and reproductive developmental stages of rice. A hierarchical cluster display generated from the average log signal values indicates differential expression profiles of genes in various tissues and development stages as given in Additional file 3, Figure S1. It shows that Os09g20220, Os01g27390, Os01g72150, Os01g37750, Os01g72160, Os10g38600 and Os10g38600 are root-specific. We also compared their expression pattern in abiotic and biotic stresses (figure S1). Our study with microarray analysis using different heavy metal stresses suggests that these GSTs are not specific to Cr (Figure 3A). To validate our microarray results we analyzed the GST activity in rice roots exposed to 100 μM Cr (VI). Higher GST activity was indeed noticed after 24 h treatment (Figure 3B).

A large number of cytochrome P450-related genes (35 nos.) were up-regulated in Cr stress. Cytochrome P450 s constitutes a large superfamily of enzymes, with 351 members in rice that are involved in the metabolism of plant biosynthetic pathways, including those for UV protectants (flavonoids, coumarins, sinapoylesters), pigments (anthocyanins), defense compounds (isoflavonoids, phytoalexins, hydroxamic acids), fatty acids, hormones (gibberellins, brassinosteroids), signaling molecules (salicylic acid, jasmonic acid, and so on), accessory pigments (carotenoids) and structural polymers (lignins) [26]. Up-regulation of Cyt450 gene family during heavy metal stress has been reported earlier, using microarray experiments, indicating their role in heavy metal detoxification [16,27]. Kawahigashi et al., [28] reported transgenic rice transformed with genes encoding human cytochrome P450 monooxygenases CYP1A1, CYP2B6, and CYP2C19 were more tolerant to various herbicides than nontransgenic Nipponbare rice, owing to enhanced metabolism by the introduced P450 enzymes. However, one of the big challenges is to define the range of functions for individual P450 proteins that show altered expression during Cr-stress.

Numerous genes related to the peroxidase family were down-regulated in Cr stress. It suggests that the thylakoid membranes are susceptible to lipid peroxidation caused by chromium stress. It is presumed that the down regulation of peroxidases may be due to chromium stress-induced damage to thylakoid membranes caused by lipoxygenases leading to increase in lipid peroxidation as evidenced by higher MDA content in root tissue (Figure 1B). The disruption of the chloroplast ultra structure and inhibition of electron transport processes due to Cr has been reported [3]. Up-regulation of a set of genes encoding peroxidases has also been reported during As stress in rice [16].

Three heat shock proteins (HSPs: Os03g16920, Os05g38530, Os03g16030) are up-regulated in Cr stress. Out of the 3 heat shock proteins, two are DnaK family proteins and one is Hsp20/alpha crystalline family protein. DnaK is the prokaryotic analogue of eukaryotic Hsp70. Out of these, two (Os03g16920, Os03g16030) have been shown to be up-regulated during As stress in rice [16]. HSPs are produced by all organisms in response to increased temperature and other stresses. These assist in the assembly, folding, and translocation of other proteins [29]. Five heat shock proteins are down-regulated in this study. Higher transcript accumulation of Hsp90-1 has been reported in tomato plants following exposure to Cr (VI) treatment [30]. Two differentially expressed dehydrins, (Os11g26760 and Os03g45280) were up- and down-regulated respectively during Cr treatment. Dehydrins are known to be produced in response to low temperature and drought stress [31,32].

Two cysteine synthase (Os06g05690, Os01g59920) and one glutathione synthetase (Os12g16200) were down-regulated in rice roots following Cr stress in our study. Glutathione synthetase is the second enzyme in the glutathione biosynthesis pathway. It catalyses the condensation of gamma-glutamylcysteine and glycine to form glutathione [33]. A large number of GSTs were up-regulated leading to reduction in the glutathione pool. Glutathione (GSH) is required to reduce Cr (VI) to Cr (III) *in vitro *[34]. Therefore, down-regulation of GSH biosynthetic genes implies that sulphate pool is reduced because of large utilization of GSH, leading to down-regulation of glutathione synthetase and cysteine synthase. One L-ascorbate oxidase homolog precursor (Os01g61160) was noticed as up-regulated in our study. This enzyme belongs to the family of oxidoreductases, participates in ascorbate metabolism [35] and might be useful in providing antioxidant pool during Cr stress in rice.

A large number of transporters were differentially expressed after challenge with Cr. Four members of sulfate transpoters were differentially expressed in our study. Out of these, two (Os01g52130, Os03g06520) were up and two (Os03g09980, Os03g09940) were down-regulated respectively during Cr stress. There are many different sulfate transporters in plants that differ in their intracellular locations, expression patterns, and kinetic properties. Sulfate can enter plants through a high-affinity sulfate transporter present in the plasma membrane [35], which may lead to enhanced production of S-rich metal-binding peptides (such as GSH, phytochelatins), thus providing metal tolerance and resulting in metal accumulation. These sulfate transporters are expressed mainly in the roots of plants and are up-regulated under sulfur-starved conditions and heavy-metal stress [16]. A transgenic mustard plant, developed using a high-affinity sulfate-transporter construct, shows altered metal tolerance and accumulation [36].

Five MATE-efflux family proteins (Os03g37470, Os06g29844, Os10g20350, Os03g37490, Os10g20350) were up-regulated on challenge with Cr. Plant MATE are localized in both vacuoles and the plasma membrane. They transport secondary metabolites and xenobiotics probably through H^+^-exchange. The rice genome possesses 59 MATE orthologs http://rice.plantbiology.msu.edu/), although their transport properties have not been elucidated. Out of the five MATEs up-regulated during Cr stress, one (Os10g20350) has been repoted to be up-regulated during As stress in rice [16]. Nawrath et al. [37] reported that the *Arabidopsis *MATE gene EDS5 is involved in the salicylic-acid-dependent signaling cascade, which is triggered by pathogens and exposure to UV light. Analysis of the potential metabolic networks from our microarray studies also indicates the possible role of salicylic-acid-dependent signaling pathway in Cr stress.

Ten ABC transporter family proteins (Os01g50100, Os01g50100, Os01g50100, Os08g30770, Os08g30740, Os09g39910, Os09g39910, Os06g38950, Os01g07870, and Os04g13220) were up-regulated in Cr stress. Polypeptides encoded by ABC transporter gene family transport a wide variety of substrates across extra- and intracellular membranes, including metabolic products, lipids and sterols, and drugs. The results suggest that ABC transporters might play an important role in transport of chromium into the cell. One transporter of a major facilitator family (Os08g06010), two metal cation transporter (Os07g12890, Os03g46470), one potassium transporter (Os01g70490), two multidrug resistance-associated proteins (Os04g49890, Os04g13210) were up-regulated specifically in Cr stress. One peptide transporter PTR2 (Os04g50940), One oligopeptide transporter (Os06g03560), and one POT family protein (Os01g65110) were also up-regulated. Moreover, six aquaporin proteins (Os06g12310, Os04g44060, Os01g74450, Os10g36924, Os02g41860, and Os06g22960) and six heavy metal-associated domain containing proteins (Os08g31340, Os01g20830, Os01g48710, Os01g48710, Os01g48710, and Os01g48710) were down-regulated in this study.

Thirty six transcription factors were differentially expressed following chromium stress. Among these, eight *WRKY *transcription factors (Os11g29870, NM_001049319, Os01g53260, Os01g43650, Os05g49620, Os01g53040, Os09g25060, Os11g02520) were up-regulated. At the same time, four *WRKY *transcription factors (Os03g58420, Os04g50920, Os02g47060, Os05g40070) are down-regulated after chromium exposure. These are involved in various plant processes but most notably, in coping with diverse biotic and abiotic stresses. One (Os11g29870) of the eight WRKY transcription factor up-regulated during Cr stress has been shown to be up-regulated during As stress also [16]. One helix-turn-helix protein (Os06g39240) was up-regulated in chromium stress which has also been shown to be up-regulated during other heavy metal stresses [16]. Eleven *MYB *family transcription factors (Os04g42950, Os06g45890, Os08g37970, Os12g01490, Os11g45740, Os11g01480, Os11g01480, Os07g48870, Os02g46780, Os04g49450, and Os06g01670) were up-regulated and thirteen *MYB *family transcription factors were down-regulated. Two *bZIP *transcription factor domain containing proteins (Os01g64730 and Os06g41770) were up- and four (Os02g14910, Os01g55150, Os01g11350 and Os03g21800) are down-regulated in Cr stress. In addition to the transcription factors, thirteen zinc finger proteins (Os04g32480, Os05g37190, Os12g10660, Os08g44190, Os12g10660, Os03g41390, Os12g39400, Os01g62130, Os02g52910, Os03g20870, Os03g53080, Os04g40090, and Os02g40810) were up- and thirty eight zinc finger proteins are down-regulated during chromium stress. This clearly suggests that a complex network of regulatory pathways modulates Cr-response of rice.

**Figure 4:**
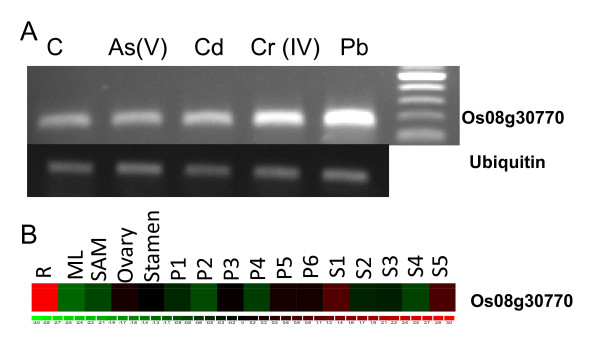
**Expression profile of one of the root-specific putative ABC transporter (Os08g30770) gene up-regulated during Cr-stress**. Semi-quantitative RT-PCR analysis during different heavy metal stresses in root (A). The rice variety IR-64 was germinated and allowed to grow for 5 d at 37°C and then transferred to Hewitt solution for growth. After 10 d of growth, seedlings of uniform size and growth were treated with 100 μM of Cr (VI), As (V), Cd, and Pb (100 μM) under standard physiological conditions. The expression profiles of ABC transporter (B) in seedling root (R), mature leaf (ML), shoot apical meristem (SAM), various stages of panicle development (P1-P6) and seed development (S1-S5). The microarray data was collected for different rice tissues/organs and developmental stages from the Gene Expression Omnibus database (GSE6893) at the National Center for Biotechnology Information and analyzed using dChip software. The color scale (representing log signal values) is shown at the bottom.

In our microarray experiments, we compared datasets for shift in expression at different developmental stages of rice and observed that 30 and 247 probe sets up- and down-regulated respectively were root specific during Cr-stress (Additional files 4 and 5, Table S3 and S4). Functional analyses suggests that the up-regulated genes include genes for GST glutathione S-transferase (5), tyrosine metabolism (4), biodegradation of xenobiotics (6), MAPK signaling pathway (5), amino acid metabolism (5), lipid metabolism (4), biosynthesis of secondary metabolites (4), signal transduction (5), ABC transporters (1), energy metabolism (2) and carbohydrate metabolism (4). The down-regulated genes are related to peroxidase (52), biosynthesis of secondary metabolites (28), lipid metabolism (24), energy metabolism (21), carbohydrate metabolism (40), amino acid metabolism (24), biodegradation of xenobiotics (27), membrane transport (13), MAPK signaling pathway (16), cell growth and death (20), signal transduction (24), cellular processes (22), metabolism of cofactors and vitamins (17), folding, sorting and degradation (6) etc. The results indicate that Cr (VI) triggers toxicity within 24 h of exposure, leading to differential expression of a large number of genes required in cellular function. This was further supported by the 7 d old seedling data where growth of the rice seedlings was significantly reduced after 100 μM Cr (VI) treatment. According to root specific analysis, members of GST gene family are up-regulated during heavy metal stress, which might help in heavy metal detoxification and is considered to be an important antioxidant involved in the cellular defense against toxicants. Only one root specific ABC transporter (Os08g30770) was up-regulated during chromium stress, which might help in the transport of chromium (Cr IV) inside the cell. The uptake of Cr (VI) is through carriers used for the uptake of essential metals for plant metabolism. The pathway of Cr (VI) transport is an active mechanism [38]. We studied the expression profile of this transporter in different heavy metal stresses and found that this gene is not only expressed in Cr (VI) but also in Pb-stress (Figure 4A). It may be possible that Pb and Cr (VI) are transported through this transporter. We also studied their expression in various vegetative and reproductive developmental stages of rice and found that this gene is root specific (Figure 4B).

Large numbers of genes related to MAPK signaling pathway are down-regulated during chromium stress. Mitogen-activated protein (MAP) kinases are serine/threonine-specific protein kinases that respond to extracellular stimuli (mitogens) and regulate various cellular activities, such as gene expression, mitosis, differentiation, proliferation, cell survival/apoptosis and are involved in cell response to growth factors [39]. The MAP kinases and a large number of genes related to amino acid metabolism are down-regulated during Cr stress. These might lead to reduced growth of plants.

Cross talk between the Cr (VI)-stress responses and abiotic stress has also been explored which revealed a high degree of similarity. Specifically, cold, draught and salt stress was found to induce many of the same genes as did chromium stress. Plant response to chromium and abiotic stresses is very similar, with both disrupting the ion and osmotic homeostasis of the plant, thus the signaling pathways would be expected to be similar. Expression of several NAC domain-containing proteins, WRKY transcription factors, cytochrome P450 and basic region/Leu Zipper (bZIP) also got induced similar to what was observed during abiotic stress [14]. There is plenty of evidence that MAPK cascade plays a crucial role in various biotic and abiotic stress responses and in hormone responses that include ROS signaling [15]. Apart from this, the role of GSH-dependent detoxification reactions is well established in our study, as a large number of GSTs are upregulated under chromium stress.

### Metabolic shift following Cr (VI) stress

Metabolites in rice roots exposed to 100 μM Cr (VI) for 24 h analyzed mainly by GC-MS and NMR. Assignment of the compounds was done comparing the GC-MS profile and ^1^H spectra of reference compounds together with Biological Magnetic Resonance Data Bank http://www.bmrb.wisc.edu/metabolomics/ and wherever necessary, by spiking with appropriate internal standards. 2 D COSY spectra was also extensively used to resolve the complexity of the overlapping/interfering spectral regions to identify the exact molecule in the extract (Additional file 6, Figure S2). The content of several metabolites including lactate, fructose, uracil and alanine (Table 3) increased following exposure to Cr stress. Proline accumulated 3-fold in comparison to control. This increase in proline content is lower in comparison to spectorophotometric measurement (Figure 1C). This might be because spectrophotometric analysis is not very sensitive method to study proline content and measurements were done on the basis of fresh weight basis where as in GCMS/NMR it is dry weight basis. However, both the data sets provided increase in proline content. Proline is a compatible osmolyte, and plays an important role in adaptation to osmotic stress [22,23,40]. Protection of structural and functional integrity of M4 lactate dehydrogenase by proline has been reported [41]. In plants, proline is synthesized not only from glutamate but also from arginine/ornithine. In our limited metabolites profiling, ornithine content also increased suggesting ornithine may be used as substrate for enhanced biosynthesis of proline. As ornithine is formed from arginine, its catabolic transformation into proline depends on the allosteric regulation of arginase by proline [42]. Our results on the higher content of ornithine suggest the significance of urea cycle in contributing to the accumulation of proline in rice roots under Cr (VI) stress. However, increased expression of the genes related to proline biosysthesis from ornithine was not observed in microarray data. This may be because of our more stringent criteria set for microarray analysis or modulation in post-transcriptional modifications of polypeptides involved in different processes. Increase in alanine and uracil content by Cr stress may be related to each other as uridine and uracil work as precursors of alanine during salt stress in *Bruguiera sexangula *cells [43]. The uracil salvage machinery in nucleotide metabolism observed in salt stressed cells may be closely related to the salt tolerance of mangrove plants due to reduction in the consumption of energy and substrates required for pyrimidine nucleotide synthesis. Similar situation might be operating during Cr stress in rice.

**Table 3:** Metabolic profiling of rice root during Cr (VI) stress

Metabolite	MS/NMR signals	Control mg/g db	Cr mg/g db	
^a^Myristic acid ME	242 (M^+^),143,87,74,44	0.50 ± 0.07	0.10 ± 0.02	Hexane
	
^a^Pentadecanoic acid ME	256(M^+^),87,74,44	0.85 ± 0.12	0.10 ± 0.03	
	
^a^Palmitic acid ME	270(M^+^), 143, 87, 74,43	4.58 ± 0.52	2.33 ± 0.07	
	
^a^Linoleic acid ME	294(M^+^),149,81,67,44	1.74 ± 0.22	4.89 ± 0.12	

^a^Ergo Sterol	472,382,343,73,44	0.27 ± 0.05	0.12 ± 0.02	EtOAc
	
^a^Stigma Sterol	484,394,255,83,44	0.45 ± 0.03	0.18 ± 0.02	
	
^a^β-Sitosterol	486,396,357,129,75,44	0.30 ± 0.07	0.16 ± 0.08	
	
^a^2-Hydroxy Propanoic acid (TMS)_2_	219(M^+^-CH_3_),147, 117,73 (Me_3_Si**^.^**)	0.93 ± 0.09	1.21 ± 0.04	
	
^a^Glycerol-(TMS)_3_	293(M^+^-CH_3_),218,205,147,117,73(Me_3_Si**^.^**)	0.71 ± 0.10	0.30 ± 0.02	
	
^a^Benzoic acid-TMS	194(M^+^),179(M^+^-CH_3_),135,105,73(Me_3_Si**^.^**)	0.14 ± 0.12	0.16 ± 0.03	
	
^a^Succinic acid-(TMS)_2_	247(M^+^-CH_3_),147,73(Me_3_Si**^.^**)	0.69 ± 0.08	0.41 ± 0.02	
	
^a^Malic acid- (TMS)_3_	335(M^+^-CH_3_), 233,147,73(Me_3_Si**^.^**)	0.10 ± 0.03	0.21 ± 0.01	
	
^a^β-Hydroxy β- Methayl Glutaric acid- (TMS)_3_	363(M^+^-CH_3_),273,247,231,147,73(Me_3_Si**^.^**)	0.18 ± 0.02	0.44 ± 0.05	
	
^a^4-Hydroxy Benzoic acid- (TMS)_2_	282(M^+^),267(M^+^-CH_3_),223,193 (M^+^-Me_3_SiO), 73(Me_3_Si**^.^**)	0.21 ± 0.07	0.33 ± 0.02	
	
^a ^Suberic acid- (TMS)_2_	303(M^+^-CH_3_),286,217,187,147,73(Me_3_Si**^.^**)	0.21 ± 0.03	2.10 ± 0.12	
	
^a^Azelaic acid-(TMS)_2_	332(M^+^),317(M^+^-CH_3_),292,73(Me_3_Si**^.^**)	3.31 ± 0.13	2.28 ± 0.15	
	
^a^4-Hydroxy Cinnamic acid-(TMS)_2_	308 (M^+^),293 (M^+^-CH_3_),249,219,179,73(Me_3_Si**^.^**)	1.61 ± 0.12	2.10 ± 0.08	

^b^Alanine	δ1.48, δ 3.77	0.37 ± 0.03	1.19 ± 0.05	Water
	
^b^Choline	δ 3.19, δ 3.52	0.25 ± 0.03	0.91 ± 0.06	
	
^a^D-Fructose- (5 TMS, MeOX_I_)	364, 307, 217,147,103,73(Me_3_Si**^.^**)	0.81 ± 0.07	1.61 ± 0.08	
	
^b^GABA	δ 1.91, δ 2.29, δ 3.01	0.66 ± 0.05	1.66 ± 0.09	
	
^a ^D-galactose	554(M^+^-CH_3_), 319, 217, 205, 147, 73(Me_3_Si**^.^**)	2.10 ± 0.10	4.87 ± 0.22	
	
^a^D-gluconic acid	613(M^+^-CH_3_), 333, 292, 217, 147, 73(Me_3_Si**^.^**)	0.63 ± 0.06	1.89 ± 0.09	
	
^b^Lactate	δ 1.33, δ 4.11	0.16 ± 0.02	1.09 ± 0.08	
	
^a^Myo-inositol	612(M^+^), 507, 318, 305, 217, 147, 73(Me_3_Si**^.^**)	0.22 ± 0.02	0.55 ± 0.05	
	
^b^Ornithine	δ 1.6-1.8	1.04 ± 0.05	3.33 ± 0.42	
	
^a^L-proline	273(M^+^), 258(M^+^-CH_3_), 230, 156, 147, 73(Me_3_Si**^.^**)	0.35 ± 0.02	1.12 ± 0.03	
	
^b^Phenyl alanine	δ 7.3-7.45	0.45 ± 0.03	0.88 ± 0.05	
	
^c^Uracil	δ 5.95, δ 7.75	0.45 ± 0.04	1.39 ± 0.60	
	
^c^Valine	δ 0.98, δ 1.04, δ 2.25	0.54 ± 0.05	1.44 ± 0.08	

^a^Indentified and quantified by GC-MS, ^b ^Identified and quantified by NMR.

Higher accumulation of various fatty acids was observed in Cr exposed rice roots. Cellular fatty acid composition is the result of a sum of complex phenomena, required for maintaining optimal viability of against external challenges. The biosynthesis of a reactive target, such as linoleic acid and its subsequent peroxidation by the radicals generated by cells after stress exposure prevents major damage to cellular DNA [44]. Our results showed that the content of linoleic acid (C18:2) was highest in rice root under Cr (VI) stress. The results agree with the known common effect of stress on the increase in unsaturation level of the fatty acids in plants [45]. The possibility of the increase of unsaturation through linoleic acid in more tolerant plants has been reported earlier [46]. Stigmasterol, β-sitosterol and campesterol are the most common phytosterols and their content was modulated during stress conditions. Salinity increased linoleic (18:2) and linolenic (18:3) acids and stigmasterol, but decreased palmitoleic (16:1) and oleic (18:1) acids and sitosterol in broccoli roots [45]. During Cr stress in rice, the level of both stigmasterol and sitosterol decreased in the roots.

**Figure 5:**
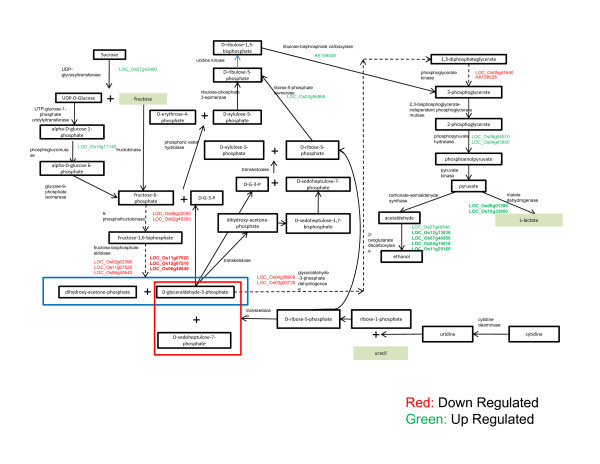
**Altered sucrose degradation pathway using Plant MetGenMAP software **http://bioinfo.bti.cornell.edu/cgi-bin/MetGenMAP/home.cgi**constructed on the basis of differentially expressed genes and metabolite content alteration**. The rice variety IR-64 was germinated and allowed to grow for 5 d at 37°C and then transferred to Hewitt solution for growth. After 10 d of growth, seedlings of uniform size and growth were treated with 100 μM of Cr (VI) under standard physiological conditions. The microarray was performed using one-cycle target labeling and control reagents. The hybridization data were analyzed using dCHIP software. Metabolites in rice roots exposed to 100 μM Cr (VI) for 24 h analyzed mainly by GC-MS and NMR.

Simultaneous analysis of microarray and metabolite content using PlantMetGenMAP software http://bioinfo.bti.cornell.edu/cgi-bin/MetGenMAP/home.cgi suggested that sucrose degradation pathway was modulated in Cr stress response (Figure 5). Three main fermentation pathways are active in plants during stress response: ethanol, lactic acid, and a plant specific pathway which produces alanine from glutamate, pyruvate and uracil. These operate as a rescue mechanism when respiration is arrested. Their expression was enhanced under Cr stress.

### Metabolic networks derived from transcriptome expression profiles

**Figure 6:**
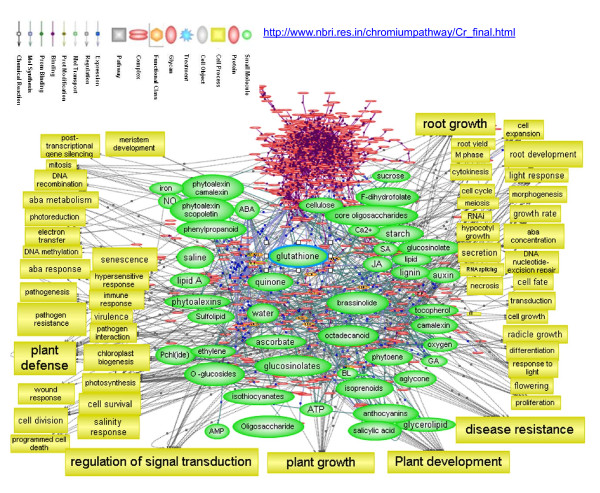
**Functional network predicted from the genes expressed differentially during Cr (VI) stress in rice (For detail pathway please visit **http://www.nbri.res.in/chromiumpathway/Cr_final.html**)**. The rice variety IR-64 was germinated and allowed to grow for 5 d at 37°C and then transferred to Hewitt solution for growth. After 10 d of growth, seedlings of uniform size and growth were treated with 100 μM of Cr (VI) under standard physiological conditions. The microarray was performed using one-cycle target labeling and control reagents (Affymetrix, USA) using 5 *μ*g RNA The hybridization data were analyzed using dCHIP software. The differentially expressed transcripts ranked by the magnitude of the moderated t-statistic determined from the statistical analysis were analyzed for cellular pathway and transcriptome interactions using the program Pathway Studio (Ariadne Genomics, USA). The pathway diagram was further filtered to show the proteins involved in cellular processes associated with the Cr response network.

The derivatisation of potential metabolic networks from microarray data is a very useful method for maximizing information [16,40]. To identify the potential changes in cellular functions, transcriptome interaction networks of differentially regulated data sets in the array have been analyzed using the software Pathway Studio (Ariadne Genomics). The pathway analysis shows that chromium-stress response influences the pathways related to signal transduction, plant growth, plant development, disease resistance, plant defense, root growth, pathogenesis and photosynthesis (Figure 6). The pathway analysis also indicates that abscisic acid metabolism, glutathione metabolism and ascorbate metabolism are affected during chromium stress. Our analysis suggests that chromium affects mainly cell growth, cell division and growth rate. By clicking on any of the biological objects within the pathway, more detailed information for each object can be obtained (for further information and detailed pathway files, please visit http://www.nbri.res.in/chromiumpathway/Cr_final.html). The nodes show the predicted metabolomes that may modulate molecular functions. The results are indicated of a vast reservoir of information which needs to be utilized validated systematically for understanding detailed mechanisms involved in the chromium stress response of rice.

### Identification of over-represented motifs in promoters of differentially regulated genes during chromium stress

As described in materials and methods, the promoters of the up-regulated, down-regulated and the total rice genes were analysed for significant abundance of the 6, 7 and 8nt motifs. The lift, support and confidence criteria used were described by Doi et al., [47]. This analysis was done in two steps, the first was based on the frequency (motifs were counted only for the presence in the promoters) and the second on the occurrence (number of times a motif occurs in a given promoter region). In the first step, custom perl script was used to calculate frequency of all the motifs in up-regulated, down-regulated and total rice promoters. The motifs having lift greater than or equal to one (11747 & 15523 from up-regulated and down-regulated promoter respectively) were selected for further analysis. In the second step, custom perl script was used to calculate the occurrence of 11747 motifs in chromium specific up-regulated promoters (599) and all rice promoters (66710). The occurrence counts of the motifs in test data set were subtracted from the control dataset to overcome the effect of test dataset in control dataset. Similar analysis was done for 15523 motifs of the down-regulated dataset. The lift based on the occurrence was calculated for 11747 and 15523 motifs. The motifs were filtered on lift greater than or equal to two (≥2). The result gave 151 and 808 motifs as over-represented in up-regulated and down-regulated promoters respectively. To further strengthen the analysis, stringent criteria of selecting motifs with greater than or equal to 100 occurrences in test dataset was adopted. Using these criteria, 14 and 13 motifs (Cr specific motifs) were selected for up-regulated and down-regulated dataset respectively. To check whether the Cr specific motifs were indeed significantly over represented in the promoter regions, occurrence of these motifs in whole genome sequence was carried out. In the background of whole genome the lift value for these 14 (Table 4) and 13 (Table 5) motifs were significantly high in the test dataset. Though no functional role has been assigned to any of the motifs, but all of these are present as promoter motifs in the Database of orthologus promoters (*doop.abc.hu/*). The motifs of up-and down-regulated datasets were classified in different clusters on the basis of their occurrence in promoters of different genes (Additional files 7 and 8, Figure S3 and S4).

**Table 4:** Putative motifs predicted in promoters of genes up-regulated during chromium stress

Motifs	Occurrence	Lift	Motif Logo
AGCTAGC	536	2.15	
	
CTAGCTA	523	2.11	
	
TAGCTAG	514	2.17	
	
GCTAGCT	483	2.10	
	
TAGCTAC	123	2.15	
	
AGCTAGCT	113	2.47	

TCGATCG	441	2.23	
	
CGATCGA	418	2.12	
	
TCGATCA	141	2.08	

GTCAAAC	243	2.10	
	
CGGTCAA	118	2.31	

ACGTACG	109	2.19	
	
CGTACGT	103	2.09	

GCTAAGC	116	2.03	

**Table 5:** Putative motifs predicted in promoters of genes down-regulated during chromium stress

Motif	Occurrence	Lift	Motif Logo
TTATCC	787	2.07	
	
CTTATC	701	2.26	
	
CTTATCC	216	3.46	
	
CCTTATC	175	3.07	
	
TCTTATC	125	2.13	
	
TTATCCT	124	2.11	
	
TTATCCA	118	2.03	
	
CCTATCC	117	2.67	
	
CTATCCA	110	2.75	
	
TATCCTC	108	2.65	

GCCCATG	118	2.12	

GATAAGG	112	2.45	

CGCGAGC	104	2.03	

### Clusters of up-regulated datasets

The promoters containing the Cr specific motifs were used for functional clustering. The promoters were clustered according to the presence of various motifs. Five clusters for the up and down-regulated genes were generated by k-means clustering and functional annotation was carried out using GO annotation

Cluster 1 of the up-regulated promoters had an over-representation of genes related to transcription factor activity, DNA binding, catalytic activity and response to stress (Figure S3a). Four motifs (AGCTAGC, CTAGCTA, TAGCTAG and AGCTAGCT) were highly represented in this set. Two other motifs (GCTAGCT and TCGATCG) were also present in these genes, though at a lower frequency. The second cluster had motifs TCGTCGA and CGATCGA (Additional file 7, Figure S3b). The genes in this cluster belonged to functional categories indicative of catalytic activity, response to stress and secondary metabolite processes. Cluster 3 included genes related to response to stress (Additional file 7, Figure S3c). The other members in the cluster included transferases, hydrolases and proteins with catalytic activity. However no distinct motifs were identified as characteristic of this cluster. Cluster 4 has a clear over representation of the motif GCTAGCT (Additional file 7, Figure S3d). There were few genes in this cluster, most of those were related to response to stress. Genes for transporters, hydrolases and protein binding activity were high in this group. Cluster 5 had an over representation of the motifs AGCTAGC and TAGCTAG (Additional file 7, Figure S3e). The genes in this cluster were related to signal transduction, response to stress, secondary metabolites, kinases, hydrolases and catalytic activity. In silico analysis of proximal promoter of one of the root specific putative ABC transporter (Os08g30770) contains 3 motifs (GTCAA; CGATC; TAGCA). These motifs are present in a large number of up-regulated genes (Table 4).

### Clusters of down-regulated datasets

Cluster 1 has an over-representation of the motifs TTATCC, CTTATC, CTTATGC and CCTTATC (Additional file 8, Figure S4a). The genes in this cluster were mainly related to translation and structural molecules. Tatematsu et al [48] reported that the promoters of genes down-regulated after main stem decapitation in *Arabidopsis*, were enriched for TTATCC motif that resembles the sugar-repressive element [49]. No functional role has been assigned to the other motifs. Cluster 2 has a clear over-representation of CTTATC motif (Additional file 8, Figure S4b). The genes in this cluster were mainly related to hydrolase activity. Over-representation of GCCCATG motif was observed in cluster 3 (Additional file 8, Figure S4c). The genes in this cluster were mainly related to response to stress and RNA binding. Cluster 4 contains motifs CCTATCC, GATAAGG, CTATCCA, TATCCTC and CGCGAGC which were present in genes related to response to stress and translation (Additional file 8, Figure S4d). Cluster5 has one motif (TATCC) which is often present in genes related to catalytic activity, nucleotide binding and hydrolase activity (Additional file 8, Figure S4e). Predominance of different motifs in the subsets of genes suggests the involvement of motif specific transcription modulating proteins in Cr stress response of rice.

## Conclusion

A significant effect on root growth being observed at 24 h at 100 μM Cr (VI) this treatment is further used for transcriptomics and metabolomics analyses. Cr (VI) treatment was associated with lipid peroxidation and an increased in proline synthesis. Transcriptomics analysis revealed that the expression of 1138 genes was up-regulated, and that of 1610 genes was down-regulated in roots by Cr (VI). Most of the genes differentially expressed under both Cr (VI) stress were related to glutathione metabolism, transport, and signal-transduction pathways. However, somewhat unexpectedly, up-regulation of phytochelatin synthase was not detected by microarray analysis suggesting that PCs are not involved in Cr (VI) detoxification. This might be due to their nonresponsive behavior to Cr (VI) stress, which is in agreement with previous report (3, 10). On the contrary, in our study it is clear that glutathione plays an important role for detoxification of Cr-stress. Simultaneous analysis of microarray and metabolite content suggested that sucrose degradation pathway was modulated in Cr stress response involving three main fermentation pathways operating as a rescue mechanism when respiration is arrested. We also analyzed presence of cis-acting elements in differentially regulated genes during Cr (VI) stress. To check whether the Cr-specific motifs were indeed significantly over represented in the promoter regions of Cr-responsive genes, occurrence of these motifs in whole genome sequence was carried out which suggests significant co-relation between differentially expressed genes and identified motifs.

## Materials and methods

### Plant Material and growth parameters

The rice variety IR-64 was germinated and allowed to grow for 5 d at 37°C and then transferred to Hewitt solution for growth. After 10 d of growth, seedlings of uniform size and growth were treated with different concentrations of Cr (0, 25, 50, 100, 250 μM) under standard physiological conditions of 16 h light (115 μmol m^-2 ^s^-1^) and 8 h dark photoperiod at 25 ± 2°C. Different concentrations of Cr were prepared using K_2_Cr_2_O_7 _(Cr VI - Merck). Length of the main root and shoot of seedlings was measured at different time intervals following Cr exposure. Roots treated with 100 μM Cr after 24 h were taken to evaluate early expressed genes subjected to Cr stress. After 10 d of growth followed by Cr treatment, shoot length, root length, fresh root weight and shoot weight were measured. All the samples were ground in liquid N_2 _and stored at -80°C.

### Metal accumulation

Harvested plants were washed thoroughly with distilled water for quantification of total metal accumulation. The roots and shoots of the plants were separated manually, dried in an oven at 80°C for one week and kept for metal analysis. For the estimation of Cr in roots and shoots of rice, 0.5g oven dried (at 70°C) grinded plants tissue were taken and digested in 3ml of HNO_3 _at 120°C for 2 h and 140°C for 4 h then filtered in 10 ml of Milli Q water and stored at 4°C till the estimation. Cr was quantified with the help of Inductively Coupled Plasma Mass Spectrometer (ICP-MS, Agilent 7500 ce) at SGS India Pvt. Ltd, Gurgaon, Haryana. The standard reference materials of metal (E-Merck, Germany) were used for the calibration and quality assurance for each analytical batch. Recovery of Cr from the plant tissue was found to be more than 95.5% as determined by spiking samples with a known amount of Cr. The detection limit for Cr was 5 μg l^-1^.

### Measurement of lipid peroxidation

Lipid peroxidation was estimated as malondialdehyde (MDA) produced using thio barbituric acid (TBA) method as described by Heath and Packer [50]. Briefly, 1g sample was homogenized in 1mL 0.5% trichloracetic acid (TCA). The homogenate was centrifuged at 19,000 × g for 20 min. The 0.5 mL supernatant was mixed with 2.5 mL TCA (20%) containing TBA (0.5%), heated in boiling water bath for 30 min and then allowed to cool rapidly in an ice bath. The supernatant was centrifuged at10, 000 × g for 10 min and the resulting supernatant was used for determination of MDA. The concentration of MDA was calculated from the absorbance at 532 nm (correction was made by subtracting absorbance at 600 nm for turbidity) by using extinction coefficient of 155mM^-1 ^cm^-1^.

### Measurement of NP-TH (non protein thiol)

NP-TH content was assayed according to Patra et al. [51] based on the affinity of 5, 5- dithiobis (2-nitrobenzoic acid) (DTNB) for -SH groups. Briefly, roots were ground in 50 mM Tris-HCl, pH 7.5, 1 mM EDTA, 0.2% (v/v) Triton X-100 (1/4; w/v). The homogenate was centrifuged at 27,000 × g for 25 min and supernatant were used for the analysis. For total -SH groups, the supernatant was mixed with DTNB (0.1 M) and methanol and the mixture was centrifuged at 12,000 × g for 10 min. The absorbance was measured at 412 nm.

For non- protein -SH group determination, the proteins were removed by TCA (12% w/v) precipitation, and after centrifugation at 12,000 × g for 10 min, thiol groups were assayed by reaction with DTNB, as mentioned above. Standard plots of L-cystein (5-40 μM ml^-1^) were used for determination of non- protein -SH group.

### Measurement of proline

Proline was determined following Bates et al. [52]. Briefly, tissue suspended in 3% sulfosalicylic acid was centrifuged at 4000 × g for 10 min to remove cell debris. To 2 ml supernatant, ninhydrin (2 ml) was added with glacial acetic acid (2 ml) and incubated at boiling temperature for 1 h. The mixture was extracted with toluene, and proline was quantified spectrophotometrically at 520 nm from the organic phase.

### GST activity

Activity of GST in the tissue samples were measured by adopting the protocol of Habig et al. [53]. Fresh plant tissue (0.5 g) was ground on ice cold mortar in potassium phosphate buffer (0.2 M; pH 7.0), homogenized and the resulting homogenate centrifuged at 4°C for 5 min at 15000 rpm. In the assay, 1 mM CDNB (1-chloro-2,4-dinitrobenzene) and 6 mM glutathione (GSH) were used as substrates. The GST in the sample catalyses the conjugation of CDNB to glutathione producing S-(2,4-dinitrophenyl) glutathione, and enzyme activity was monitored spectrophotometrically at 340 nm.

### Total RNA extraction and transcriptome analysis

Total RNA was extracted from the treated rice roots using the QIAGEN RNeasy Plant Maxi Kit (QIAGEN, MD). The yield and RNA purity were determined spectrophotometrically (NanoDrop, Wilmington, DE) and by formaldehyde-agarose gel electrophoresis. The microarray was performed using one-cycle target labeling and control reagents (Affymetrix, USA) using 5 *μ*g RNA. Affymetrix Gene Chip Rice Genome Arrays (Gene Expression Omnibus platform accession no. GPL2025) were used for microarray analysis. Target preparation, hybridization to arrays, washing, staining, and scanning were carried out according to manufacturer's instructions (Affymetrix, USA). Affymetrix Gene Chip Operating Software 1.2.1 was used for washing and scanning in Fluidics Station 450 (Affymetrix, USA) and Scanner 3300 (Affymetrix, USA), respectively. Three independent replicated experiments were carried out for all the treatments. The hybridization data were analyzed using dCHIP software. Satisfactory image files were analyzed to generate probe intensity (.cel) files using the default settings of GCOS. The normalization of all arrays was performed following default setting of dCHIP [54]. To identify statistically significant differentially expressed genes, a combined criterion of 2-fold or more change and t test *p *value, < 0.005 was adopted. To obtain annotations for the probe sets, target sequences were from the sequence information file for the rice genome array. The target sequences were then searched using BLASTN against the TIGR rice pseudomolecules, release 6.1 http://rice.plantbiology.msu.edu/. For the genes that were differentially regulated, the gene ontology classification provided in http://bioinfoserver.rsbs.anu.edu.au/utils/GeneBins/ was used to assign genes to a hierarchical biological process using the Genebins [55]. Details of the microarray can be found at Gene Expression Omnibus (GEO; http://www.ncbi.nlm.nih.gov/projects/geo/) under accession number GSE25206.

The microarray data was collected for different rice tissues/organs and developmental stages, including germinating seedling (GS), seedling root (R), mature leaf (ML), leaf (YL; leaf subtending the shoot apical meristem), shoot apical meristem (SAM), and various stages of panicle (P1-P6) and seed (S1-S5) development (Jain et al., 2007; GSE6893). GSE7951 (expression profiling of stigma), GSE6901 (expression data for stress treatment), GSE7256 (expression data for virulent infection by *Magnaporthe grisea*), and GSE10373 (expression data for interaction with the parasitic plant *Striga hermonthica*) were selected for the analysis of probe sets that are differentially expressed in Cr-stress. The CEL files were downloaded from the Gene Expression Omnibus database at the National Center for Biotechnology Information and analyzed using dChip [54] software.

### Expression analysis by Real-Time PCR

Total RNA was extracted as per procedure given in previous section. First strand cDNA was synthesised using 5 μg purified total RNA and RevertAid First Strand cDNA synthesis Kit (Fermantas, Life Sciences, USA). Real Time PCR was performed in 25 μl for set of selected genes using Power SYBR Green PCR Master Mix (ABI, USA). List of selected genes and oligonucleotide primers (MWG, India) used for each gene are listed in the Additional file 9, Table S5. Oligonucleotide primers for rice actin gene were used as internal control for establishing equal amount of cDNA in all the reactions. The reactions were performed using the following cycle conditions, an initial 94°C for 2 min, followed by 30 cycles of 94°C for 30 s, 60°C for 30 s, and 72°C for 30 s, and the final 5 min extension at 72°C. After obtaining ct value for each reaction, the fold change was calculated by using Delta-Delta ct method.

### Pathway analysis

The differentially expressed transcripts ranked by the magnitude of the moderated t-statistic determined from the statistical analysis were analyzed for cellular pathway and transcriptome interactions using the program Pathway Studio (Ariadne Genomics, USA). The pathway diagram was further filtered to show the proteins involved in cellular processes associated with the Cr response network.

### Samples preparation for metabolom analysis

Fresh roots of rice were collected after Cr (VI) treatment and washed with MiliQ water. Adherent water was removed using blotting papers and immediately frozen in liquid nitrogen followed by crashing with mortar and pastel. Crashed tissue were lyophilized to remove moisture from the sample and kept at -20°C until use for analysis. For metabolomics investigation dried plant material was extracted with ten times of its weight of hexane. The solvent portion was collected by filtration and this procedure was repeated five more times until the hexane layer became almost colourless. Collected hexane layer was concentrated under reduced pressure and resulting sticky mass was stored at -20°C until analyzed by GC-MS. The remaining solid plant material was further extracted thrice with five fold excess (w/w) of 90% and then with 70% warm methanol-water. Volume of the extract was reduced to 1/3 rd using rotavapour and defatted with equal volume of hexane. Defatted water-methanol layer was partitioned (liquid-liquid) with equal volume of EtOAc (five times). EtOAc layers was separated and dried over sodium sulphate, concentrated to semisolid mass and stored at -20°C till GC-MS analysis. Remaining methanolic water layer was lyophilized to dryness and the resulting solid was again saved similarly for NMR and GC-MS analysis.

All the solvents used for the extraction of phytochemicals from rice root tissues were purchased from Qualigen (ExcealR). All detureted solvents for NMR were purchased from Sigma Chemical Company (USA).

### GC-MS Method

GC-MS analysis was performed using Thermo Trace GC Ultra coupled with Thermo fisher DSQ II mass spectrometers (Thermo Scientific, USA) with electron impact ionisation at 70 eV to generate mass spectra. Thermo TR50 column 30 m × 0.25 mm (polysiloxane column coated with 50% methyl and 50% phenyl groups) was used for chromatographic separation of metabolites. To prepare the sample for GC-MS analysis of non polar hexane extract, 10 mg portion was heated at 60°C for 6 h with 5 ml of methanolic sulphuric acid (5%, v/v). After cooling, the reaction mixture was diluted and vigorously shaken with 25 ml hexane and 20 ml water. Separated hexane layer was washed with 20 ml water containing 5% (w/v) sodium bicarbonate followed by equal volume of 5% (w/v) sodium chloride solution. Hexane layer was collected and concentrated using rota vapour after drying over anhydrous sodium sulphate. The resulting oily mass was dissolved in 1 ml of GC-grade *n*-hexane and 0.4 μl of the solution subjected to analysis on GC. With an initial 5-min solvent delay time at 70°C, the oven temperature was increased to 330°C at 5°C min^-1^, 5 min isocratic and cooled down to 70°C followed by an additional 5-min delay. Helium flow was maintained at 1 ml min^-1^ and split ratio was maintained at 1/60. The resulting GC-MS profile was analyzed using WILLY and NIST mass spectral library http://www.nist.gov/data/nist1a.htm and by matching the chromatogram with Supelco FAME mixture and whenever possible, with appropriate standards. For the GC-MS analysis of other than hexane extracts, the trimethylsilyl (TMS) derivative of the sample was prepared. Approximately 5 mg of the sample was suspended in 40 μl of the solution of methoxylamine hydrochloride in pyridine (20 μg ml^-1^). The mixture was shaken for 2 h at 37°C before adding 70 μl of MSTFA. Shaking was continued for another 30 mins followed by GC-MS analysis. The quantification of identified metabolites was done using percentage peak area of each metabolite.

### NMR analysis

^1^H NMR spectra of the hexane and aqueous methanolic extracts were obtained on Bruker Biospin Avance 400 MHz NMR spectrometer (Bruker Biospin, USA) using a 5 mm broad band inverse probe head, equipped with shielded z-gradient accessories. One-dimensional ^1^H-NMR spectral analyses of hexane extracts were carried out using one-pulse sequence by dissolving samples in 500 ml deuterated chloroform taken in 5-mm NMR tubes. A reusable sealed capillary tube containing 30 ml of 0.375% trimethylsilyl phosphate (TSP) in deuterium oxide was inserted into the NMR tube before recording the spectra. TSP served as chemical shift reference as well as internal standard for quantitative estimation. Typical parameters for both the extractions were: spectral width: 6000 Hz; time domain data points: 32 K. For quantification purpose the effective flip angle of 45° was used, optimized and standardized instead of 90° using total relaxation delay of 7.73 s for complete recovery of the magnetization by taking consideration of our earlier studies on amino acids [56] so that the quantified results are precise; spectrum size: 32 K points; and line broadening for exponential window function: 0.3 Hz. To confirm the assignments, two-dimensional (2D) correlation spectroscopy (COSY) was carried out using the Bruker's standard pulse program library. The spectral widths of COSY were 6,000 Hz in both dimensions, and 512 t_1 _increments for each t_1_. 16 transients using 2.5 s relaxation delays were added with 2048 complex data. The phase-sensitive data were obtained by the time proportional phase incrementation (TPPI) method. The resulting data were zero-filled up to 1024 in t_1 _dimension and were weighted with 90° squared sine window functions in both dimensions prior to double Fourier transformation. The experiments were performed with a spectral width of 6,000 Hz in F_2 _dimension and 24,000 Hz in F_1 _dimension, 400 t_1 _increments. For each t_1_, 96 transients using 1.5 s relaxation delay was added with 2048 complex data points. The assignments were further reinstated based on the existing literature values obtained in lettuce leaves by Sobolev et al. [57].

Each of the characteristic peaks of specific metabolite was integrated with respect to signals of known amount of TSP and metabolites were quantified by following equation [58]:

$$\text{Weight of metabolites}=\frac{\text{Mw metabolite}}{\text{Mw TSP}}\times \frac{\text{No}\text{. of H TSP}}{\text{No of H metabolite}}\times \frac{\text{Integral area of metabolie}}{\text{Integral area of TSP}}\times \text{wt}.\text{ of TSP}$$

### Identification of over-represented motifs in promoters of differentially regulated genes during Cr (VI) stress

Microarray data sets of cold, drought and salinity stress (Gene Expression Omnibus database at the National Center for Biotechnology Information under the series accession numbers GSE6901) were compared with data set following chromium stress. Chromium-specific rice up-regulated genes (599) and down-regulated genes (388) were selected. One kb upstream regions from these genes were extracted from rice genome database (TIGR) and used as the test dataset. As control dataset one kilobase upstream regions from all rice promoters (66710) were used To find novel cis-regulatory elements specific to chromium stress, a database was constructed which consist of all possible combinations of 6, 7 & 8 base lengths. Perl Script was written to count the number of genes in which a given motif was present in the dataset of up-regulated, down-regulated and all rice promoters (66710). The above result was filtered for motifs with minimum of 10 and 20 counts for down and up-regulated genes respectively in the test dataset. Lift, Support and Confidence values were calculated for the filtered dataset [47].

The up and down-regulated genes were clustered as per to the presence of the different motifs. For each of the up and down regulated sets, 5 K-means cluster group were created using the MeV software [47]. The GO annotation for the rice loci in each of the clusters were downloaded from the Rice Genome Annotation Project http://rice.plantbiology.msu.edu/.

## Authors' contributions

SD, PM, SC and SD performed the experiments; DC, SB, SM and MHA performed the data analysis; DC, PKT, RDT and RT wrote the manuscript; all of the authors contributed to the research design, discussed the results and commented on the manuscript.

## Supplementary Material

Additional File 1 **Table S1**. List of genes up-regulated during Cr (VI) stress in rice. Click here for file

Additional File 2 **Table S2**. List of genes down-regulated during Cr (VI) stress in rice. Click here for file

Additional File 3 **Figure S1**. Expression profiles of differentially expressed GST during 100 μM Cr (VI) in rice roots. The microarray data from different rice tissues/organs and developmental stages including seedling root (R), mature leaf (ML), leaf (YL; leaf subtending the shoot apical meristem), shoot apical meristem (SAM), and various stages of panicle (P1-P6) and seed (S1-S5) development. The data were taken from (GSE6893), GSE7951 (expression profiling of stigma), GSE6901 (expression data for stress treatment), GSE7256 (expression data for virulent infection by *Magnaporthe grisea*), and GSE10373 (expression data for interaction with the parasitic plant *Striga hermonthica*) were selected for analysis of GST-probe sets that are differentially expressed in Cr-stress. Click here for file

Additional File 4 **Table S3**. List of root-specific genes up-regulated during Cr (VI) stress in rice. Click here for file

Additional File 5 **Table S4**. List of root-specific genes down-regulated during Cr (VI) stress in rice. Click here for file

Additional File 6 **Figure S2**. The COSY spectrum of aqueous fraction of rice roots. Click here for file

Additional File 7 **Figure S3 a-e**. K-means clustering and GO annotations of genes up-regulated during chromium stress. The clustering was done according to the presence of the motifs identified in this study. a(i) cluster1 a(ii) GO annotation of cluster1. b(i) cluster2 b(ii) GO annotation of cluster2 c(i) cluster3 c(ii) GO annotation of cluster3 d(i) cluster4 d(ii) GO annotation of cluster4 e(i) cluster5 e(ii) GO annotation of cluster5 Click here for file

Additional File 8 **Figure S4 a-e**. K-means clustering and GO annotations of genes down-regulated during chromium stress. The clustering was done according to the presence of the motifs identified in this study. a(i) cluster1 a(ii) GO annotation of cluster1. b(i) cluster2 b(ii) GO annotation of cluster2 c(i) cluster3 c(ii) GO annotation of cluster3 d(i) cluster4 d(ii) GO annotation of cluster4 e(i) cluster5 e(ii) GO annotation of cluster5 Click here for file

Additional File 9 **Table S5**. List of primers used for RT-PCR analysis of Cr (VI) stress responsive genes in rice roots. Click here for file
