# Supplementary figures and images for: Transcriptomic and metabolomic shifts in rice roots in response to Cr (VI) stress
Source: BMC Genomics. 2010 Nov 20;11:648. doi: 10.1186/1471-2164-11-648 (PMC3224690; doi:10.1186/1471-2164-11-648)

## Slide 1
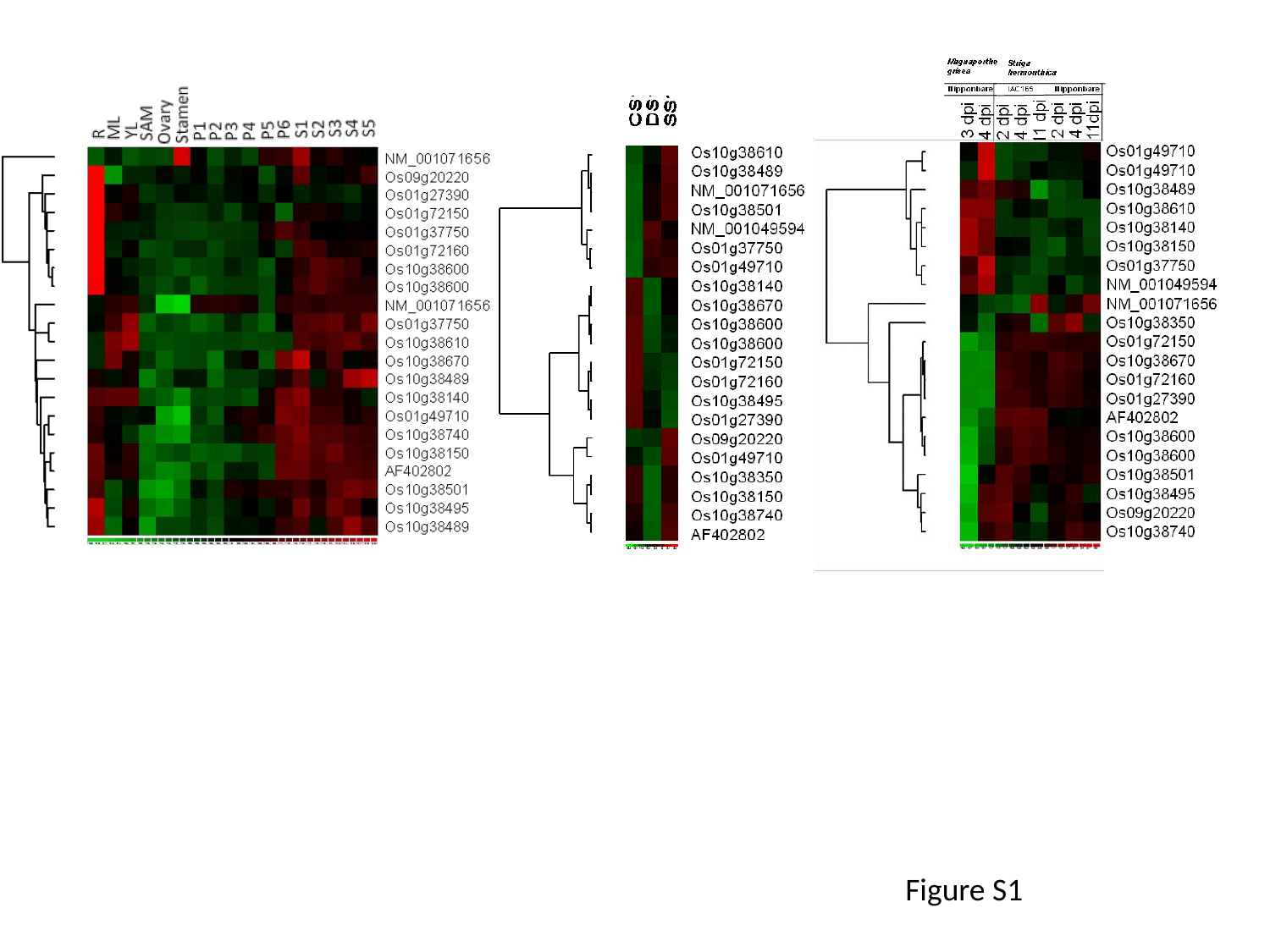

Figure S1

Supplement: Additional File 3 — Figure S1. Expression profiles of differentially expressed GST during 100 μM Cr (VI) in rice roots. The microarray data from different rice tissues/organs and developmental stages including seedling root (R), mature leaf (ML), leaf (YL; leaf subtending the shoot apical meristem), shoot apical meristem (SAM), and various stages of panicle (P1-P6) and seed (S1-S5) development. The data were taken from (GSE6893), GSE7951 (expression profiling of stigma), GSE6901 (expression data for stress treatment), GSE7256 (expression data for virulent infection by Magnaporthe grisea), and GSE10373 (expression data for interaction with the parasitic plant Striga hermonthica) were selected for analysis of GST-probe sets that are differentially expressed in Cr-stress. [file 1471-2164-11-648-S3.PPT]

## Slide 1
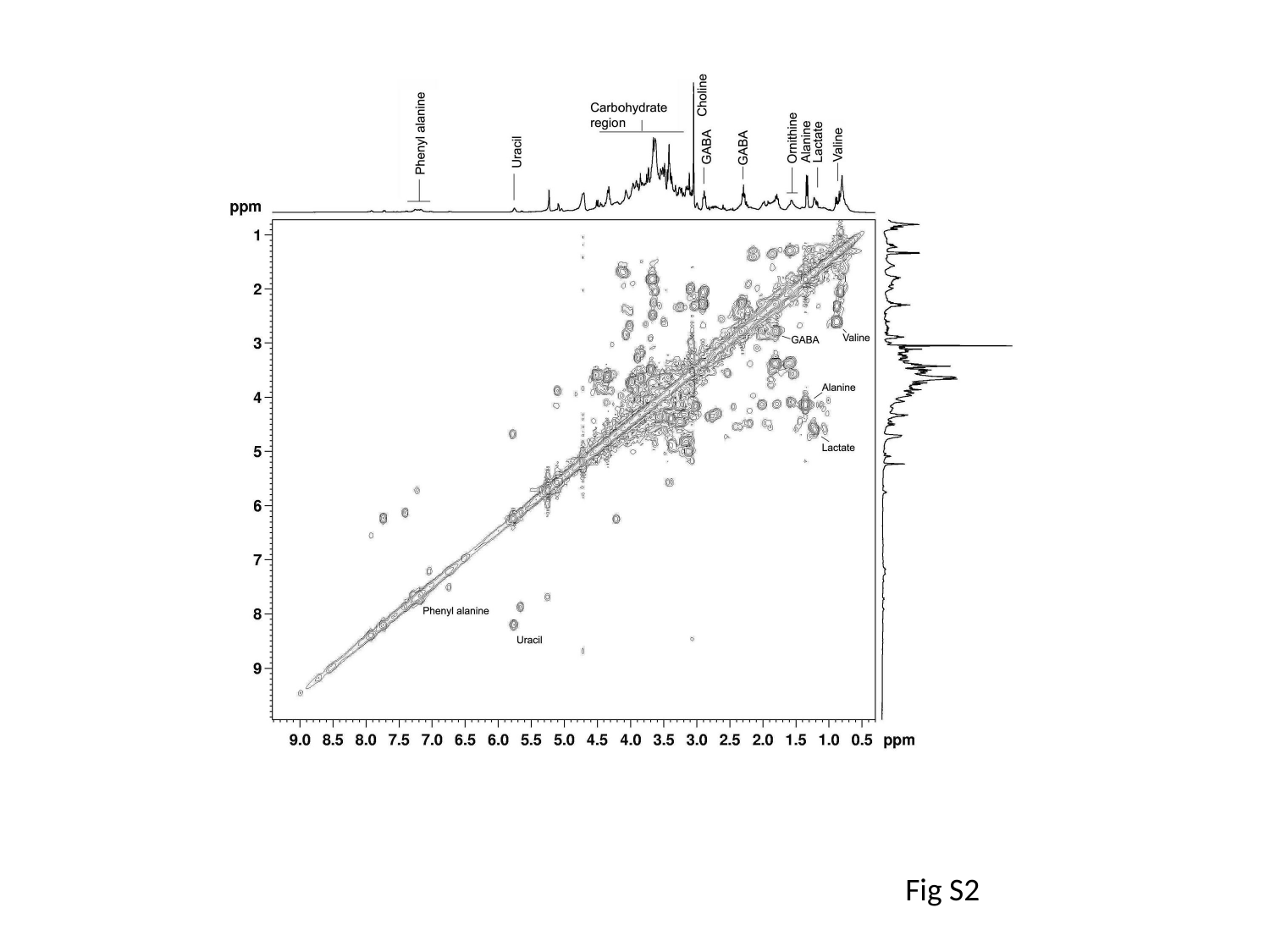

Fig S2

Supplement: Additional File 6 — Figure S2. The COSY spectrum of aqueous fraction of rice roots. [file 1471-2164-11-648-S6.PPT]
