# Supplementary material for: Transcriptomic and metabolomic shifts in rice roots in response to Cr (VI) stress
Source: BMC Genomics. 2010 Nov 20;11:648. doi: 10.1186/1471-2164-11-648 (PMC3224690; doi:10.1186/1471-2164-11-648)
Supplement: Additional File 4 — Table S3. List of root-specific genes up-regulated during Cr (VI) stress in rice. [file 1471-2164-11-648-S4.DOC]

Table S3 Root-specific genes up-regulated during Cr-stress

| **Probe ID** | **Locus ID** | **Description** | **FC** |
| --- | --- | --- | --- |
| OsAffx.19506.1.S1_at  Os.56946.1.S1_at  Os.11218.1.S1_at  Os.27713.1.A1_at  Os.9427.1.S1_at  OsAffx.25894.1.S1_x_at  OsAffx.7332.1.S1_at  Os.18981.1.S1_at  Os.27964.1.S1_at  Os.18695.1.S1_at  Os.49030.1.A1_s_at  Os.9565.1.S1_x_at  OsAffx.14448.1.S1_at  Os.9218.1.S1_at  Os.2376.1.S1_at  Os.56365.1.S1_at  OsAffx.24011.1.S1_at  Os.50620.1.S1_at  Os.54203.1.S1_at  Os.19632.1.S1_at  OsAffx.15138.1.S1_at  Os.52054.1.S1_at  Os.5941.1.S1_at  Os.33885.1.S1_at  Os.40000.1.S1_x_at  OsAffx.27513.1.S1_s_at  OsAffx.27066.1.S1_at  Os.11251.1.S1_at  OsAffx.31049.1.S1_at | LOC_Os12g05510  LOC_Os11g14910  LOC_Os07g44440  LOC_Os11g37900  LOC_Os10g05020  LOC_Os04g06760  LOC_Os11g37940  LOC_Os07g18120  LOC_Os03g29150  LOC_Os08g30770  LOC_Os09g20220  LOC_Os12g18360  LOC_Os04g56930  LOC_Os12g24390  LOC_Os03g17480  LOC_Os03g19370  LOC_Os01g72150  LOC_Os12g44190  LOC_Os09g32260  LOC_Os03g46470  LOC_Os05g47660  LOC_Os01g72160  LOC_Os01g53040  LOC_Os01g06220  LOC_Os10g38600  LOC_Os06g08640  LOC_Os05g28770  LOC_Os10g04020  LOC_Os11g18870 | conserved hypothetical protein  NADP-dependent oxidoreductase, putative, expressed  Unknown  3-ketoacyl-CoA synthase, putative, expressed  cytochrome P450, putative, expressed  hypothetical protein  WIP2 - Wound-induced protein precursor, expressed  aldehyde oxidase, putative, expressed  NAD dependent epimerase/dehydratase family protein, putative, expressed  ABC transporter, ATP-binding protein, putative, expressed  glutathione S-transferase, putative, expressed  Unknown  Beta-fructofuranosidase, insoluble isoenzyme 5, putative, expressed  Retrotransposon protein, putative, unclassified, expressed  IN2-1 protein, putative, expressed  CPuORF4 - conserved peptide uORF-containing transcript, expressed  glutathione S-transferase, putative, expressed  ATPase 3, putative, expressed  Unknown  metal cation transporter, putative, expressed  lipid phosphatase protein, putative, expressed  glutathione S-transferase, putative, expressed  OsWRKY14 - Superfamily of TFs having WRKY and zinc finger domains, expressed  gibberellin receptor GID1L2, putative, expressed  glutathione S-transferase GSTU6, putative, expressed  transferase family protein, putative, expressed  GCRP9 - Glycine and cysteine rich family protein precursor, expressed  OsFBX356 - F-box domain containing protein, expressed  osFTL11 FT-Like11 homologous to Flowering Locus T gene; contains Pfam profile PF01161: Phosphatidylethanolamine-binding protein | 25.67  5.1  2.8  2.64  4.57  4.2  3.9  2.49  11.51  9.3  24.96  2.81  7.89  2.49  15.26  6.57  14.75  3.33  2.59  9.35  4.37  4.19  2.76  5.95  7.18  7.4  2.84  8.22  2.54 |
