# Supplementary material for: Transcriptomic and metabolomic shifts in rice roots in response to Cr (VI) stress
Source: BMC Genomics. 2010 Nov 20;11:648. doi: 10.1186/1471-2164-11-648 (PMC3224690; doi:10.1186/1471-2164-11-648)
Supplement: Additional File 5 — Table S4. List of root-specific genes down-regulated during Cr (VI) stress in rice. [file 1471-2164-11-648-S5.DOC]

Table S4. Root-specific genes down-regulated during Cr-stress

| **Probe ID** | **Locus ID** | **Description** | **FC** |
| --- | --- | --- | --- |
| Os.11556.1.S1_at  Os.21817.1.S1_at  Os.49220.1.S1_at  Os.7989.1.S1_at  Os.27918.1.A1_at  OsAffx.24981.1.S1_at  Os.52222.1.S1_at  OsAffx.13127.1.S1_x_at  Os.49217.1.S1_at  OsAffx.13951.1.S1_at  OsAffx.24544.1.S1_at  Os.53639.1.S1_at  OsAffx.25753.1.S1_at  Os.2260.1.S1_at  Os.7112.1.S1_at  OsAffx.31996.1.S1_x_at  OsAffx.6404.1.S1_at  Os.55417.1.S1_at  Os.54019.1.S1_at  OsAffx.13016.1.S1_x_at  OsAffx.31574.2.S1_at  OsAffx.14631.1.S1_at  OsAffx.24232.2.S1_s_at  Os.56910.1.S1_s_at  OsAffx.17724.1.S1_at  Os.54289.1.S1_x_at  Os.9967.2.S1_x_at  Os.20535.1.S1_at  Os.6838.1.S1_at  Os.51558.2.S1_at  Os.22995.1.S1_at  Os.24965.1.S1_at  OsAffx.8033.1.S1_at  OsAffx.19632.1.S1_at  Os.50154.1.S1_at  OsAffx.28099.1.S1_at  Os.9945.1.S1_at  Os.49676.1.S1_at  Os.36709.1.S1_x_at  Os.6763.1.S1_at  OsAffx.19332.1.S1_at  Os.820.1.S1_at  OsAffx.16709.2.S1_s_at  Os.2957.1.S1_at  Os.56034.1.S1_at  Os.5185.1.S1_at  OsAffx.25008.1.S1_s_at  Os.52845.1.S1_at  Os.7793.1.S1_at  OsAffx.20067.1.S1_s_at  OsAffx.31420.1.S1_at  Os.22241.1.S1_s_at  Os.56176.1.S1_at  Os.6649.1.S1_at  Os.49614.1.S1_at  Os.5588.1.S1_s_at  Os.51270.1.S1_at  OsAffx.22107.1.S1_x_at  Os.55806.1.S1_at  Os.53788.1.S1_at  Os.53033.1.S1_at  Os.26941.1.A1_at  Os.21842.1.S1_at  Os.5052.1.S1_at  Os.53438.1.S1_at  OsAffx.18050.2.S1_at  Os.5905.1.S1_at  Os.57344.1.S1_at  Os.9708.1.S1_at  Os.57170.1.S1_at  Os.12664.1.S1_at  OsAffx.26662.1.S1_x_at  Os.55056.1.A1_at  Os.27789.1.A1_at  Os.26719.1.S1_at  Os.4965.1.S1_at  Os.24971.1.A1_s_at  OsAffx.28946.1.S1_at  Os.20265.1.S1_x_at  OsAffx.32095.1.S1_at  Os.27945.1.A1_at  OsAffx.16029.1.S1_x_at  Os.29768.1.S1_at  Os.23174.1.S1_s_at  OsAffx.26423.1.S1_at  OsAffx.10957.1.S1_x_at  Os.57309.1.S1_at  OsAffx.29831.1.S1_s_at  Os.50119.1.S1_at  Os.50125.1.S1_at  OsAffx.985.7.S1_x_at  Os.10171.1.S1_at  OsAffx.23246.1.S1_x_at  OsAffx.14956.1.S1_at  Os.20290.1.S1_at  Os.47802.1.A1_at  Os.21193.1.S1_at  Os.51246.1.S1_at  OsAffx.21077.1.S1_at  Os.7087.1.S1_at  Os.54195.1.S1_at  OsAffx.24414.1.S1_at  Os.53995.1.S1_at  Os.8855.1.S1_at  Os.2615.1.S1_at  Os.49844.1.S1_at  Os.15298.1.S1_at  Os.10539.1.S1_at  Os.2373.1.S1_at  OsAffx.2917.1.S1_s_at  Os.43666.1.S1_at  Os.11554.1.S1_at  Os.20265.2.S1_at  Os.18683.1.S1_at  OsAffx.26878.1.S1_at  Os.49750.1.S1_at  Os.21894.1.S1_at  Os.31788.1.S1_at  OsAffx.18687.1.S1_x_at  OsAffx.17976.1.S1_at  OsAffx.19633.1.S1_at  Os.10029.1.S1_at  Os.51655.2.S1_x_at  Os.56824.1.S1_at  Os.36470.1.S1_at  Os.51657.1.S1_at  OsAffx.6250.1.S1_at  OsAffx.24868.1.S1_at  OsAffx.30816.2.S1_at  Os.8682.2.S1_x_at  Os.27440.1.S1_at  Os.54004.1.S1_at  Os.7659.1.S1_s_at  OsAffx.5550.1.S1_at  Os.28427.2.S1_x_at  OsAffx.28364.1.S1_at  Os.10018.1.S1_at  Os.410.1.S1_at  Os.48328.1.S1_at  Os.53357.1.S1_at  Os.10578.1.A1_at  OsAffx.30196.1.S1_at  Os.50672.1.S2_x_at  Os.53709.1.S1_at  Os.42977.1.S1_at  Os.10435.1.S1_at  Os.8582.1.S1_at  Os.37328.1.S1_at  OsAffx.25512.1.S1_s_at  OsAffx.28981.1.S1_at  OsAffx.2707.1.S1_at  Os.28773.1.S1_at  Os.10760.1.S1_at  Os.9318.1.S1_at  Os.10270.1.S1_at  Os.54056.1.S1_at  Os.18744.1.S1_at  OsAffx.22888.1.S1_at  Os.7088.1.S1_at  OsAffx.2974.1.S1_s_at  Os.10013.1.S1_at  Os.1478.1.S1_at  Os.2961.1.S1_at  Os.56899.1.S1_at  Os.18734.1.S1_at  Os.54165.1.S1_at  Os.46669.1.A1_at  Os.9180.1.S1_at  Os.18598.1.S1_at  Os.32360.1.A1_at  Os.36663.1.S1_at  Os.6021.1.S1_at  Os.54044.1.S1_at  OsAffx.24314.1.S1_at  Os.47380.1.S1_at  Os.54147.1.S1_at  OsAffx.32059.1.S1_at  Os.49120.1.S1_at  Os.51097.1.S1_at  OsAffx.31733.1.S1_at  Os.23059.1.S1_s_at  OsAffx.14183.1.S1_at  Os.47370.1.S1_s_at  Os.48060.1.A1_at  OsAffx.30816.1.S1_s_at  Os.47592.1.A1_at  Os.27017.1.A1_at  Os.5063.1.S1_at  Os.6645.1.S1_at  OsAffx.28204.1.S1_at  Os.52574.1.S1_at  OsAffx.27695.1.S1_at  Os.53342.1.S1_at  Os.52158.1.S1_at  Os.56849.1.S1_x_at  OsAffx.19414.2.S1_at  Os.54324.1.S1_at  Os.33676.2.S1_at  Os.53691.1.S1_at  Os.27932.1.S1_at  Os.55958.1.S1_at  Os.18566.1.S1_at  Os.11212.1.S1_at  OsAffx.24308.1.S1_s_at  Os.23084.1.S1_at  Os.26775.1.A1_at  OsAffx.16990.1.S1_at  Os.47370.1.S1_at  OsAffx.26128.1.S1_at  Os.11085.1.S1_at  Os.42977.2.S1_x_at  OsAffx.6325.1.S1_at  OsAffx.21988.1.S1_at  Os.50602.1.S1_at  Os.21832.1.S1_at  Os.55718.1.S1_at  Os.2965.1.S1_at  OsAffx.2917.1.S1_at  Os.57531.1.S1_at  Os.47601.1.A1_at  Os.51831.1.S1_at  Os.28429.2.S1_x_at  OsAffx.7199.1.S1_at  Os.6792.1.S1_at  OsAffx.14583.1.S1_at  Os.51112.1.S1_at  Os.23942.1.A1_at  OsAffx.29165.1.S1_at  Os.56031.1.S1_at  Os.28994.1.S1_at  Os.32667.1.S1_at  Os.52733.1.S1_at  Os.46725.1.S1_at  Os.15219.1.S1_at  Os.9276.1.S1_s_at  Os.27183.1.S1_at | LOC_Os07g48030  LOC_Os01g21650  LOC_Os09g12970  LOC_Os02g20360  LOC_Os03g05530  LOC_Os03g05530  LOC_Os06g41480  LOC_Os03g30420  LOC_Os03g14300  LOC_Os04g24319  LOC_Os02g32770  LOC_Os04g37650  LOC_Os03g58420  LOC_Os06g05690  LOC_Os03g05500  LOC_Os12g32960  LOC_Os09g26170  LOC_Os08g15420  LOC_Os05g15690  LOC_Os03g25150  LOC_Os12g04320  LOC_Os05g11130  LOC_Os02g13710  LOC_Os07g48390  LOC_Os09g15950  LOC_Os06g10930  LOC_Os07g34580  LOC_Os03g06000  LOC_Os10g40510  LOC_Os01g59840  LOC_Os06g07250  LOC_Os01g15540  LOC_Os10g33060  LOC_Os12g12590  LOC_Os05g01790  LOC_Os06g44410  LOC_Os03g43410  LOC_Os04g55610  LOC_Os01g48710  LOC_Os01g48710  LOC_Os11g42430  LOC_Os01g19020  LOC_Os07g44280  LOC_Os03g25330  LOC_Os03g57410  LOC_Os07g48390  LOC_Os03g07510  LOC_Os03g07510  LOC_Os11g31570  LOC_Os12g41090  LOC_Os11g41150  LOC_Os11g41150  LOC_Os07g17010  LOC_Os01g74110  LOC_Os04g33520  LOC_Os03g09980  LOC_Os02g54180  LOC_Os01g11760  LOC_Os05g20930  LOC_Os04g10434  LOC_Os05g41090  LOC_Os05g07420  LOC_Os10g38670  LOC_Os06g36450  LOC_Os06g38580  LOC_Os02g56370  LOC_Os06g32990  LOC_Os03g17150  LOC_Os06g35630  LOC_Os03g41130  LOC_Os07g35810  LOC_Os04g59020  LOC_Os02g48200  LOC_Os06g20150  LOC_Os10g22570  LOC_Os03g59440  LOC_Os04g29960  LOC_Os07g47660  LOC_Os01g41120  LOC_Os12g39450  LOC_Os04g59020  LOC_Os07g01400  LOC_Os01g03620  LOC_Os08g14200  LOC_Os04g41970  LOC_Os01g07850  LOC_Os04g19960  LOC_Os09g13400  LOC_Os02g36414  LOC_Os04g57860  LOC_Os01g59550  LOC_Os03g01300  LOC_Os01g14520  LOC_Os05g34325  LOC_Os07g01410  LOC_Os12g36840  LOC_Os10g27980  LOC_Os11g11780  LOC_Os12g20400  LOC_Os02g12690  LOC_Os11g05390  LOC_Os02g25020  LOC_Os06g44970  LOC_Os02g31840  LOC_Os03g01260  LOC_Os06g37750  LOC_Os03g19990  LOC_Os11g35350  LOC_Os10g40710  LOC_Os02g39620  LOC_Os01g17160  LOC_Os06g33080  LOC_Os01g41120  LOC_Os06g44280  LOC_Os05g14220  LOC_Os04g50920  LOC_Os10g31720  LOC_Os01g03630  LOC_Os11g05390  LOC_Os09g31410  LOC_Os12g12600  LOC_Os07g44550  LOC_Os08g40720  LOC_Os06g47750  LOC_Os07g15320  LOC_Os08g44830  LOC_Os09g12400  LOC_Os02g54570  LOC_Os11g04550  LOC_Os10g08780  LOC_Os01g33869  LOC_Os12g22800  LOC_Os08g38560  LOC_Os07g36750  LOC_Os05g12640  LOC_Os07g09520  LOC_Os02g43410  LOC_Os05g19570  LOC_Os01g24560  LOC_Os02g34940  LOC_Os03g20420  LOC_Os09g39070  LOC_Os02g02140  LOC_Os02g36440  LOC_Os01g73720  LOC_Os03g62060  LOC_Os08g14109  LOC_Os01g72740  LOC_Os03g43400  LOC_Os08g01710  LOC_Os02g21300  LOC_Os01g14650  LOC_Os01g62110  LOC_Os02g44310  LOC_Os01g15810  LOC_Os09g29160  LOC_Os01g59570  NM_183766  LOC_Os07g01440  LOC_Os02g45890  LOC_Os10g31640  LOC_Os03g19427  LOC_Os03g25340  LOC_Os07g44590  LOC_Os03g01700  LOC_Os04g56420  LOC_Os10g31730  LOC_Os10g31680  LOC_Os03g49260  LOC_Os04g58760  LOC_Os01g27390  LOC_Os03g14150  LOC_Os05g04690  LOC_Os02g18930  LOC_Os04g46810  LOC_Os02g03900  LOC_Os12g36840  LOC_Os02g25700  LOC_Os05g07600  LOC_Os12g13720  LOC_Os12g36840  LOC_Os04g37970  LOC_Os10g31630  LOC_Os02g4408  LOC_Os11g04550  LOC_Os10g31630  LOC_Os08g14810  LOC_Os07g38550  LOC_Os07g25050  LOC_Os06g51000  LOC_Os04g41450  LOC_Os06g18880  LOC_Os09g25560  LOC_Os09g23140  LOC_Os03g22600  LOC_Os11g47150  LOC_Os08g33479  LOC_Os03g32790  LOC_Os03g05060  LOC_Os01g68580  LOC_Os12g01480  LOC_Os01g37490  LOC_Os07g18750  LOC_Os02g18540  LOC_Os03g49270  LOC_Os02g09790  LOC_Os08g14200  LOC_Os10g31630  LOC_Os04g24340  LOC_Os06g35140  LOC_Os01g73710  LOC_Os09g18450  LOC_Os08g06190  LOC_Os03g05070  LOC_Os05g44930  LOC_Os12g44320  LOC_Os03g25280  LOC_Os02g39620  LOC_Os04g48170  LOC_Os12g12720  LOC_Os02g15340  LOC_Os12g33160  LOC_Os11g25470  LOC_Os10g39260  LOC_Os05g07740  LOC_Os03g50860  LOC_Os02g35180  LOC_Os08g12840  LOC_Os05g11250  LOC_Os01g67390  LOC_Os01g12070  LOC_Os04g03980  LOC_Os10g05130  LOC_Os06g11320  LOC_Os07g07900  LOC_Os06g07100 | peroxidase precursor, putative, expressed  plant protein of unknown function domain containing protein, expressed  Unknown  tyrosine aminotransferase, putative, expressed  Unknown  nodulin, putative, expressed  vrga1, putative, expressed  cytochrome P450, putative, expressed  Unknown  jasmonate-induced protein, putative, expressed  cytochrome P450, putative, expressed  xyloglucan fucosyltransferase, putative, expressed  OsWRKY6 - Superfamily of TFs having WRKY and zinc finger domains, expressed  cysteine synthase, chloroplast/chromoplast precursor, putative, expressed  DUF260 domain containing protein, putative, expressed  LTPL148 - Protease inhibitor/seed storage/LTP family protein precursor, putative  MYB family transcription factor, putative, expressed  CSLC3 - cellulose synthase-like family C, expressed  expansin precursor, putative, expressed  transposon protein, putative, unclassified, expressed  sulfotransferase domain containing protein, expressed  cytochrome P450, putative, expressed  ethylene-responsive transcription factor TINY, putative, expressed  proline-rich protein, putative, expressed  expressed protein  xyloglucan fucosyltransferase, putative, expressed  glycosyl hydrolases family 16, putative, expressed  expansin precursor, putative, expressed  LTPL144 - Protease inhibitor/seed storage/LTP family protein precursor, expressed  Os1bglu3 - beta-glucosidase homologue, similar to G. max isohydroxyurate hydrolase, expressed  Protein GOS9, putative, expressed  Kinesin-4, putative, expressed  hcr2-5D, putative  NADP-dependent oxidoreductase, putative, expressed  expressed protein  histidine kinase, putative, expressed  OsIAA12 - Auxin-responsive Aux/IAA gene family member, expressed  Expressed protein  Metal ion binding protein, putative, expressed  heavy metal-associated domain containing protein, expressed  transporter family protein, putative, expressed  peroxidase precursor, putative, expressed  dirigent, putative, expressed  Peroxidase 66 precursor, putative, expressed  RING-H2 finger protein ATL5D, putative, expressed  proline-rich protein, putative, expressed  expressed protein  expressed protein  Expressed protein  CAMK_KIN1/SNF1/Nim1_like.38 - CAMK includes calcium/calmodulin depedent protein kinases, expressed  nitrilase-associated protein, putative, expressed  nitrilase-associated protein, putative, expressed  chalcone synthase, putative, expressed  metal cation transporter, putative, expressed  RCLEA6 - Root cap and Late embryogenesis related family protein precursor, expressed  sulfate transporter, putative, expressed  expressed protein  GDSL-like lipase/acylhydrolase, putative, expressed  ZOS5-07 - C2H2 zinc finger protein, expressed  amidase family protein, putative, expressed  CAMK_CAMK_like_CG18020d.1 - CAMK includes calcium/calmodulin depedent protein kinases, expressed  S-domain receptor-like protein kinase, putative, expressed  glutathione S-transferase, putative, expressed  ferroportin1 domain containing protein, expressed  Unknown  OsWAK20 - OsWAK receptor-like protein kinase, expressed  peroxidase precursor, putative, expressed  ZOS3-09 - C2H2 zinc finger protein, expressed  reticuline oxidase-like protein precursor, putative, expressed  expressed protein  TKL_IRAK_DUF26-ld.6 - DUF26 kinases have homology to DUF26 containing loci, expressed  integral membrane protein, putative, expressed  lectin-like protein kinase, putative, expressed  peroxidase precursor, putative, expressed  cellulase, putative, expressed  dirigent, putative, expressed  OsWAK43 - OsWAK receptor-like protein kinase, expressed  expressed protein  retrotransposon protein, putative, Ty3-gypsy subclass, expressed  Hypothetical protein  integral membrane protein, putative, expressed  peroxidase precursor, putative, expressed  multicopper oxidase domain containing protein, expressed  glycosyl hydrolases family 16, putative, expressed  endoglucanase, putative, expressed  glyoxalase family protein, putative, expressed  retrotransposon protein, putative, unclassified, expressed  Unknown  transporter family protein, putative, expressed  endoglucanase precursor, putative, expressed  senescence-induced receptor-like serine/threonine-protein kinase precursor, putative, expressed  Cortical cell-delineating protein precursor, putative, expressed  C4-dicarboxylate transporter/malic acid transport protein domain containing protein, expressed  cytochrome P450 51, putative, expressed  peroxidase precursor, putative, expressed  pathogenesis-related Bet v I family protein, putative, expressed  transmembrane amino acid transporter protein, putative, expressed  cysteine-rich receptor-like protein kinase 21 precursor, putative, expressed  Unknown  cytochrome P450, putative, expressed  transporter, major facilitator family, putative, expressed  DNA binding protein, putative, expressed  auxin efflux carrier component, putative, expressed  expressed protein  expansin precursor, putative, expressed  S-locus-like receptor protein kinase, putative, expressed  WD40-like Beta Propeller Repeat family protein, expressed  expressed protein  expansin precursor, putative, expressed  ATOZI1, putative, expressed  OsSubt1 - Putative Subtilisin homologue, expressed  peroxidase precursor, putative, expressed  retrotransposon protein, putative, Ty3-gypsy subclass, expressed  retrotransposon protein, putative, Ty3-gypsy subclass, expressed  tyrosine protein kinase domain containing protein, putative, expressed  OsWRKY37 - Superfamily of TFs having WRKY and zinc finger domains, expressed  glycine-rich cell wall structural protein 2 precursor, putative, expressed  multicopper oxidase domain containing protein, expressed  transporter, major facilitator family, putative, expressed  Os9bglu29 - beta-glucosidase homologue, similar to Os4bglu12 exoglucanase, expressed  dirigent, putative, expressed  peroxidase precursor, putative, expressed  FAD-binding and arabino-lactone oxidase domains containing protein, putative, expressed  phytosulfokine receptor precursor, putative, expressed  leaf senescence related protein, putative, expressed  ZOS8-14 - C2H2 zinc finger protein, expressed  hypothetical protein  SCP-like extracellular protein, expressed  sulfotransferase domain containing protein, expressed  expressed protein  expressed protein  expressed protein  receptor-like protein kinase 2 precursor, putative, expressed  CSLF3 - cellulose synthase-like family F; beta1,3;1,4 glucan synthase, expressed  expressed protein  expressed protein  transposon protein, putative, unclassified, expressed  expansin precursor, putative, expressed  fruit bromelain precursor, putative, expressed  OsPDIL1-3 protein disulfide isomerase PDIL1-3, expressed  alpha-N-arabinofuranosidase A, putative, expressed  thiol protease SEN102 precursor, putative, expressed  receptor protein kinase CLAVATA1 precursor, putative, expressed  transporter family protein, putative, expressed  RCLEA4 - Root cap and Late embryogenesis related family protein precursor, expressed  hydrolase, putative, expressed  retrotransposon protein, putative, Ty1-copia subclass, expressed  cytochrome P450, putative, expressed  OsIAA11 - Auxin-responsive Aux/IAA gene family member, expressed  GLTP domain containing protein, putative, expressed  transposon protein, putative, unclassified, expressed  expansin precursor, putative, expressed  expressed protein  LTPL112 - Protease inhibitor/seed storage/LTP family protein precursor, expressed  peroxidase precursor, putative, expressed  expressed protein  senescence-induced receptor-like serine/threonine-protein kinase precursor, putative, expressed  Unknown  plastocyanin-like domain containing protein, putative, expressed  sulfotransferase domain containing protein, expressed  glycine-rich cell wall structural protein 2 precursor, putative, expressed  nicotianamine synthase, putative, expressed  peroxidase precursor, putative, expressed  peroxidase precursor, putative, expressed  expressed protein  retrotransposon protein, putative, unclassified, expressed  retrotransposon protein, putative, unclassified, expressed  glycine-rich cell wall structural protein 2 precursor, putative, expressed  lipoxygenase, putative, expressed  membrane associated DUF588 domain containing protein, putative, expressed  glutathione S-transferase, putative, expressed  expressed protein  CHIT11 - Chitinase family protein precursor, expressed  calcineurin B, putative, expressed  LTPL120 - Protease inhibitor/seed storage/LTP family protein precursor, expressed  metal transporter Nramp6, putative, expressed  Pathogenesis-related protein 10, putative, expressed  Acyltransferase, putative, expressed  expressed protein  Plant PDR ABC transporter associated domain containing protein, expressed  pathogenesis-related Bet v I family protein, putative, expressed  transporter family protein, putative  expressed protein  Unknown  sulfotransferase domain containing protein  Unknown  Unknown  expressed protein  Thionin-like peptide, putative, expressed  expressed protein  TGF-beta receptor, type I/II extracellular region, putative, expressed  cadmium tolerance factor, putative  BT1 family protein, putative, expressed  endonuclease/exonuclease/phosphatase family domain containing protein, expressed  BTBN6 - Bric-a-Brac, Tramtrack, Broad Complex BTB domain with non-phototropic hypocotyl 3 NPH3 and coiled-coil domains, expressed  OsWAK122 - OsWAK receptor-like protein kinase  expressed protein  expressed protein  exostosin family domain containing protein, expressed  LTPL38 - Protease inhibitor/seed storage/LTP family protein precursor, expressed  phosphatidate cytidylyltransferase, putative, expressed  dynein light chain type 1 domain containing protein, expressed  LTPL42 - Protease inhibitor/seed storage/LTP family protein precursor, expressed  uncharacterized Cys-rich domain containing protein, putative, expressed  THION36 - Plant thionin family protein precursor, expressed  Unknown  glycosyl hydrolases family 16, putative, expressed  expressed protein  phytase, putative  MYB family transcription factor, putative, expressed  RCLEA3 - Root cap and Late embryogenesis related family protein precursor, putative  flavonol synthase/flavanone 3-hydroxylase, putative, expressed  berberine and berberine like domain containing protein, expressed  exostosin family domain containing protein, expressed  receptor-like protein kinase, putative, expressed  carbohydrate binding protein, putative, expressed  peroxidase precursor, putative, expressed  ATOZI1, putative, expressed  cytochrome P450, putative, expressed  jasmonate-induced protein, putative, expressed  no apical meristem protein, putative, expressed  RGH1A, putative, expressed  hypothetical protein  aspartic proteinase nepenthesin, putative, expressed  receptor-like protein kinase 2 precursor, putative, expressed  histidine kinase, putative, expressed  OsRR2 type-A response regulator, expressed  FabA-like domain containing protein, expressed  lachrymatory factor synthase, putative, expressed  POEI32 - Pollen Ole e I allergen and extensin family protein precursor, expressed  endoglucanase precursor, putative, expressed  flavin monooxygenase, putative, expressed  expressed protein  peptidyl-prolyl cis-trans isomerase CYP40, putative, expressed  expressed protein  RING-H2 finger protein, putative, expressed | -5.14  -15.7  -10.6  -3.15  -3.26  -3.26  -3.07  -2.93  -9.28  -4.69  -4.26  -13.25  -6.39  -2.75  -4.01  -8.2  -8.33  -3.58  -6.22  -3.4  -14.01  -2.65  -10.37  -4.48  -13.93  -4.48  -3.77  -12.28  -8.73  -6.9  -5.95  -3.48  -6.93  -11.76  -7.62  -6.5  -3.92  -2.41  -5.14  -6.52  -3.96  -3.9  -9.56  -12.74  -5.29  -8.51  -4.64  -3.82  -14.22  -11.38  -3.5  -8.29  -4.53  -2.34  -5.08  -5.23  -8.62  -14.41  -3.15  -9.88  -2.84  -13.75  -2.46  -12.11  -7.44  -4.69  -2.67  -5.77  -3.25  -3.78  -3.45  -2.83  -6.06  -3.41  -4.88  -23.09  -7.67  -30.72  -7.4  -4.66  -8.84  -5.29  -3.59  -5.34  -9  -13.09  -7.11  -20.21  -5.11  -7.35  -3.98  -7.44  -8.48  -3.2  -7.48  -3.18  -9.11  -14.43  -8.08  -12.25  -3.75  -9.82  -4.89  -3.99  -7.21  -4.43  -5.78  -13.8  -36.47  -8.9  -8.49  -6.57  -5.47  -11.84  -4.81  -4.94  -7.82  -6.06  -6.22  -5.84  -4.49  -12.49  -5.59  -18.79  -7.16  -19.12  -4.75  -2.95  -20.45  -5.11  -6.54  -5.86  -4.59  -6.86  -8.42  -7.43  -4.16  -4  -13.14  -27.5  -13.5  -4.96  -4.08  -7.93  -7.02  -13.87  -9.07  -78.87  -33.16  -2.63  -12.21  -3.52  -6.72  -8.99  -10.44  -8.32  -32.36  -19.35  -5.34  -11.28  -11.31  -2.69  -4.89  -37.74  -16.6  -13.39  -3.6  -5.1  -8.09  -6.79  -48.98  -32.24  -12.45  -3.97  -11.89  -3.59  -59.75  -11.85  -7.39  -4.73  -4.64  -2.57  -11.52  -6.35  -4.36  -4.35  -7.24  -3.75  -4.14  -6.83  -3.52  -9.98  -4.24  -3.24  -4.31  -5.41  -3.11  -8.49  -4.66  -7.33  -11.61  -7.36  -4.26  -10.12  -9.58  -18.65  -7.32  -2.98  -7.29  -3.8  -14.7  -3.04  -10.87  -5.25  -6.41  -3.72  -3.22  -3.04  -98.09  -9.57  -18.14  -4.62  -4.97  -9.63  -5.06  -3.28  -2.71  -3.36  -3.76  -3.78  -3.71  -17.74  -10.69  -10.35  -10.09  -2.57 |
