# Supplementary material for: Transcriptomic and metabolomic shifts in rice roots in response to Cr (VI) stress
Source: BMC Genomics. 2010 Nov 20;11:648. doi: 10.1186/1471-2164-11-648 (PMC3224690; doi:10.1186/1471-2164-11-648)
Supplement: Additional File 8 — Figure S4 a-e. K-means clustering and GO annotations of genes down-regulated during chromium stress. The clustering was done according to the presence of the motifs identified in this study. a(i) cluster1 a(ii) GO annotation of cluster1. b(i) cluster2 b(ii) GO annotation of cluster2 c(i) cluster3 c(ii) GO annotation of cluster3 d(i) cluster4 d(ii) GO annotation of cluster4 e(i) cluster5 e(ii) GO annotation of cluster5 [file 1471-2164-11-648-S8.PPT]

## Slide 1
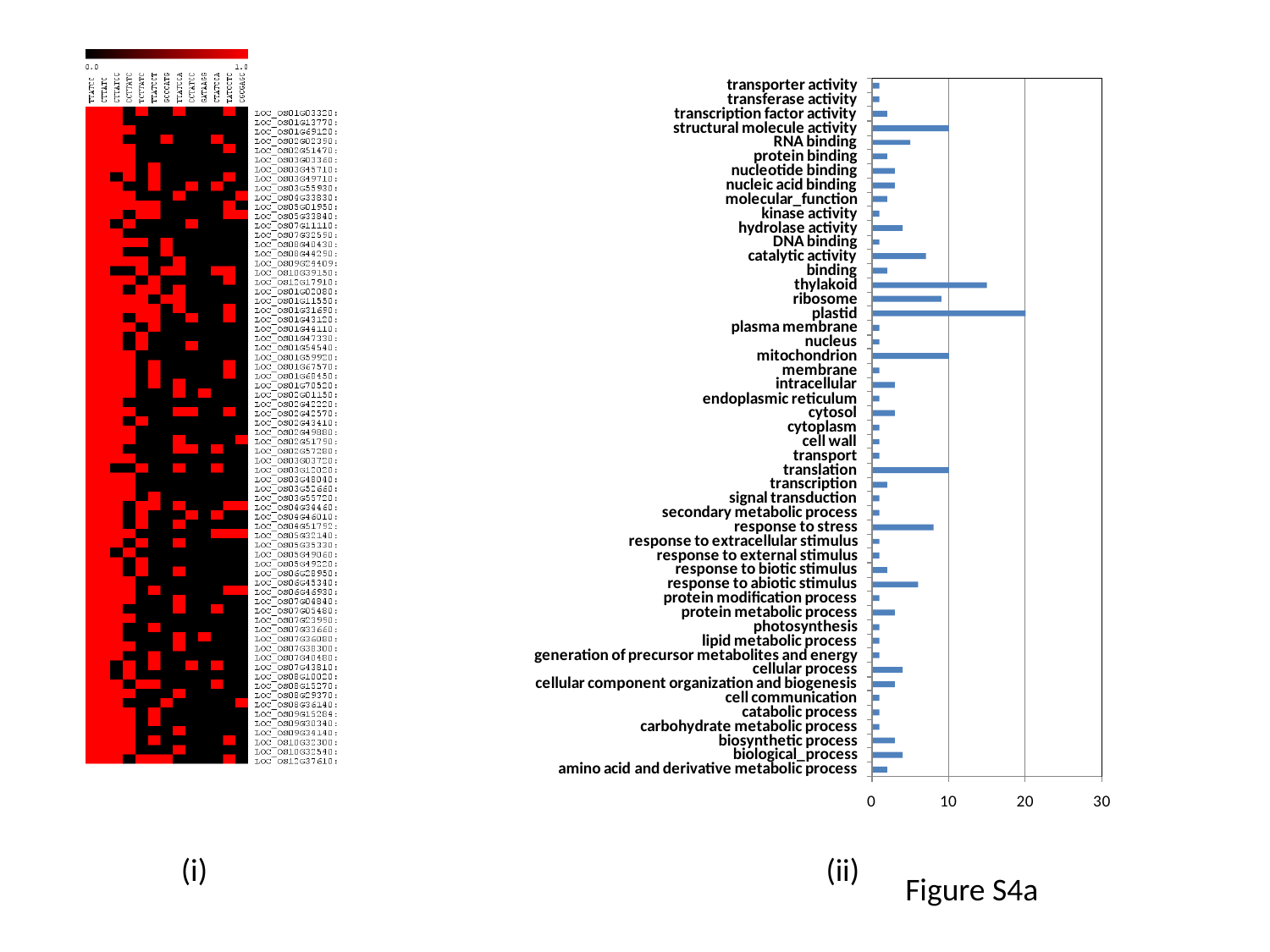

(i)
(ii)
Figure S4a

## Slide 2
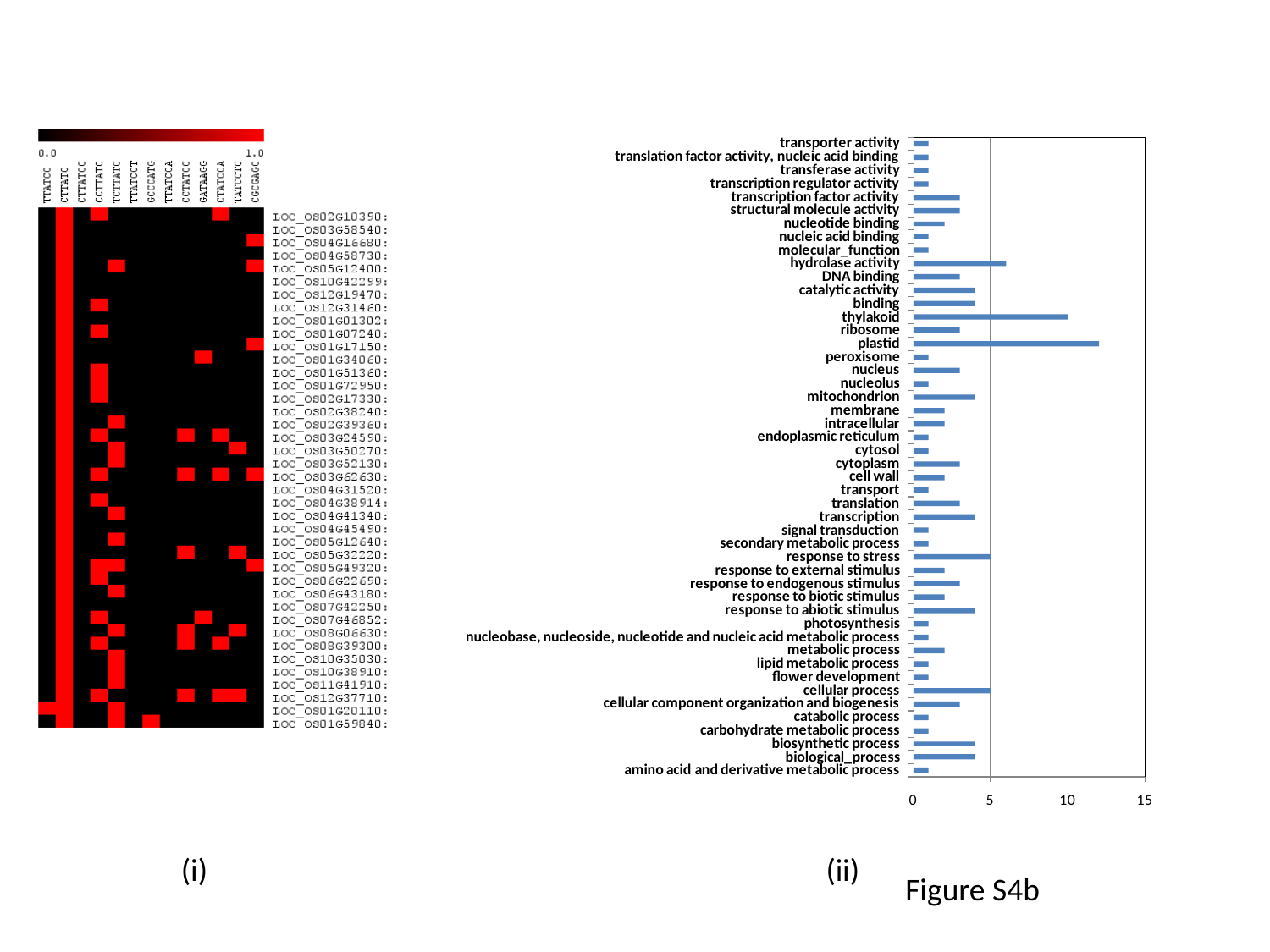

(i)
(ii)
Figure S4b

## Slide 3
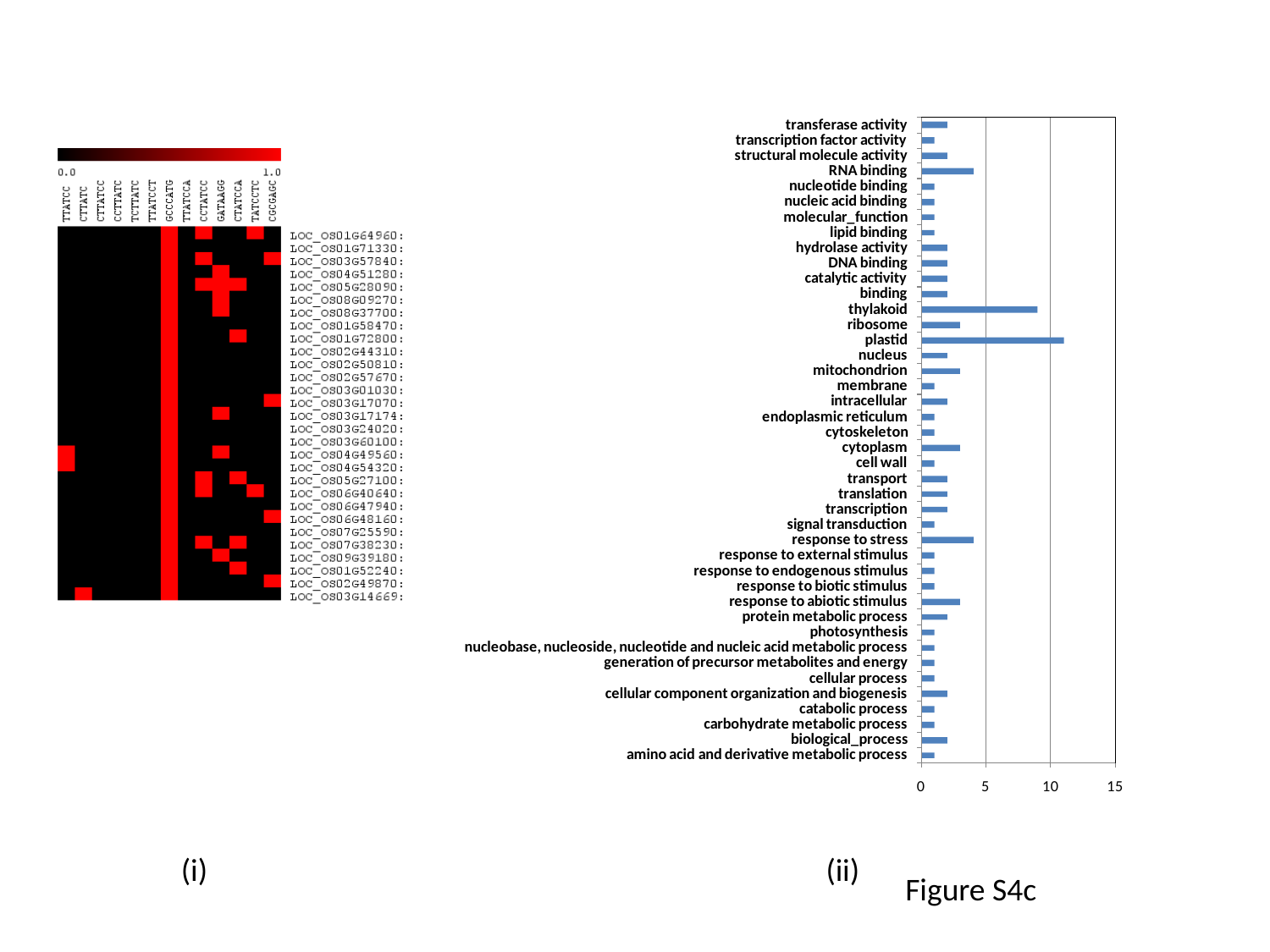

(i)
(ii)
Figure S4c

## Slide 4
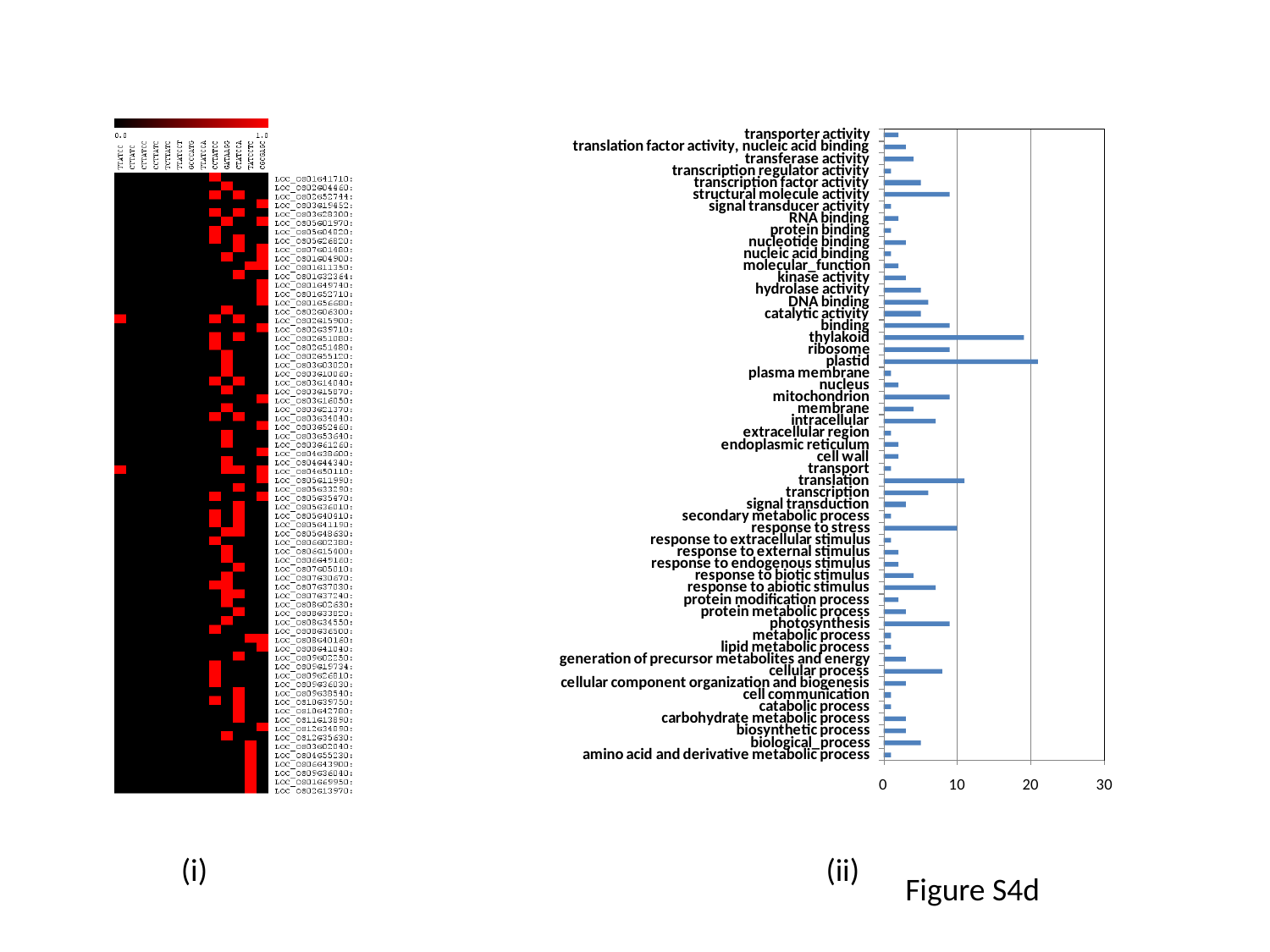

(i)
(ii)
Figure S4d

## Slide 5
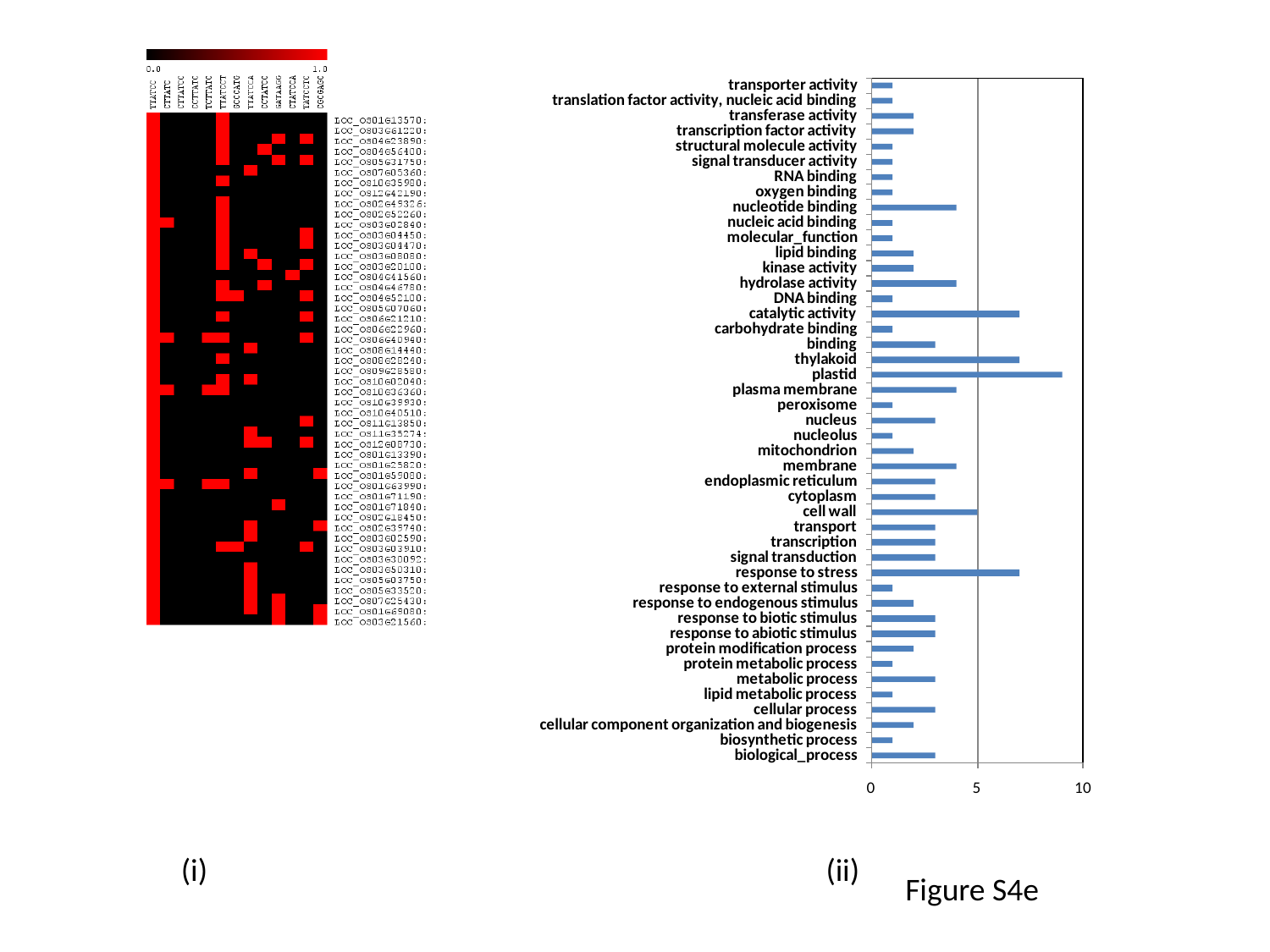

(i)
(ii)
Figure S4e
