# Supplementary material for: Transcriptomic and metabolomic shifts in rice roots in response to Cr (VI) stress
Source: BMC Genomics. 2010 Nov 20;11:648. doi: 10.1186/1471-2164-11-648 (PMC3224690; doi:10.1186/1471-2164-11-648)
Supplement: Additional File 9 — Table S5. List of primers used for RT-PCR analysis of Cr (VI) stress responsive genes in rice roots. [file 1471-2164-11-648-S9.DOC]

Table S5: List of primers used for RT-PCR analysis of Cr (VI) stress responsive genes in rice roots.

| **Locus ID** | **Primers** | |
| --- | --- | --- |
| LOC_Os07g33780 | F: 5’-ATTCCATGATTGGCTATGAGTG-3’  R: CAAGAACGGCCGCAAGCATTTC-3’ | |
| LOC_Os08g30770 | F: 5’-AGACGTTTTCCATGCAGTTGAGAAG-3’  R: 5’-AGACGTTTTCCATGCAGTTGAGAAG-3’ | |
| LOC_Os01g50100 | F: 5’-AAGATACTGCTACTTGATGAG-3’  R: 5’-GTGTGCCACTACAATGGTAGTC-3’ | |
| LOC_Os04g13210 | F: 5’-CGAAGCAACATTGATCCAATTG-3’  R: 5’-CACTATCAGCCACTAGAGCATC-3’ | |
| LOC_Os06g03560 | F: 5’-GGACTTAGGCACTTATTCAGC-3’  R: 5’-GTTCTGGCCTGGGAATGTCTTG-3’ | |
| LOC_Os04g50940 | F: 5’-ATGCCGCAGTACTTCATGCTG-3’  R: 5’-GCCAGCGACGTGCAGATGCTC-3’ | |
| LOC_Os01g52130 | F: 5’-GTCACTTCTGTCGACAACTCTG-3’  R: 5’-TGACGTATCCAGACAGCACCAG-3’ | |
| LOC_Os02g36030 | F: 5’-GCCATGGTGATCTTCAACGCG-3’  R: 5’-CCGCGGAAATCGATCGCCGTAG-3’ | |
| LOC_Os01g43740 | F: 5’-CCAAGGCGACCAAGACCAATC-3’  R: 5’-AAGTATCACGCATAACGCCATC-3’ | |
| LOC_Os03g12500 | F: 5’-TGCTGTTCGGCTACCAGCCGTG-3’  R: 5’-GTACACGTATTGCAGCAGCTTC-3’ | |
| LOC_Os01g41820 | F: 5’-AACACGAGACACATGGAAGTG-3’  R: 5’-CTTAATAGCAGCATGGTCCATG-3’ | |
| LOC_Os03g55240 | F: 5’-TGCTGGGCACGCTGATCCAGTG-3’  R: 5’-ACATGGCCTCCAACGGCACGAC-3’ | |
| LOC_Os06g45960 | F: 5’-TGGAGGCACAAACTATGAATTC-3’  R: 5’-CCATCTGGTGTAACACTAGGAG-3’ | |
| LOC_Os01g37750 | F: 5’-CAGGATCGTGGACGCGGACAG-3’  R: 5’-CCGCCGCGATGAGGCGTTCGAG-3’ | |
| LOC_Os10g38350 | F: 5’-CAAGCCCATCTCCGAGTCCATG-3’  R: 5’-GAACAGCTTGTCGTCGACGTAG-3’ | |
| LOC_Os10g38495 | F: 5’-CTCGCGGCGGTGGAGACGCTG-3’  R: 5’-GATGAGGCCACCAAGCGCGACG-3’ | |
| LOC_Os10g38610 | F: 5’-CATCGACGACAAGCTACTGGCG-3’  R: 5’-CAGAACGTCCACCGCGACGAAC-3’ | |
| LOC_Os01g49710 | F: 5’-GACCTACTTCGACAGCACGTTC-3’  R: 5’-CCTTGGAGCACTCCACGAACG-3’ | |
| LOC_Os01g72150 | F: 5’-AGCTATCTGCGAATCACTTGTC-3’  R: 5’-ACGGCAACGTGCACTTGTGATC-3’ | |
| LOC_Os03g16920 | F: 5’-GAGATCGAGCGGATGGTGCAG-3’  R: 5’-GCGCATGTTGTACGCGTAGTTC-3’ | |
| LOC_Os05g38530 | F: 5’-GAGATCGAGAAGATGGTGCAG-3’  R: 5’-GTTGCGCATGTTGTAGGCGTAG-3’ | |
| LOC_Os03g16030 | | F: 5’-CAGCGGCAAGTTCCTCCGCAG-3’  R: 5’-GCCGGTAACCTGGATGGAC-3’ |
